# Molecular orientation-dependent energetic shifts in solution-processed non-fullerene acceptors and their impact on organic photovoltaic performance

**DOI:** 10.1038/s41467-023-37234-0

**Published:** 2023-04-04

**Authors:** Yuang Fu, Tack Ho Lee, Yi-Chun Chin, Richard A. Pacalaj, Chiara Labanti, Song Yi Park, Yifan Dong, Hye Won Cho, Jin Young Kim, Daiki Minami, James R. Durrant, Ji-Seon Kim

**Affiliations:** 1 Department of Physics and Centre for Processable Electronics, Imperial College London, London, SW7 2AZ UK; 2 Department of Chemistry and Centre for Processable Electronics, Imperial College London, London, W12 0BZ UK; 3 School of Energy and Chemical Engineering, Ulsan National Institute of Science and Technology (UNIST), Ulsan, 44919 Republic of Korea; 4 Graduate School of Carbon Neutrality, Ulsan National Institute of Science and Technology (UNIST), Ulsan, 44919 Republic of Korea; 5 CSE team, Innovation Center, Samsung Electronics, Co. Ltd., 1 Samsungjeonja-ro, Hwaseong-si, Gyeonggi-do 18448 Republic of Korea; 6 SPECIFIC IKC, Department of Materials, University of Swansea, Bay Campus, Swansea, SA1 8EN UK; 7 Present Address: Department of Physics, The Chinese University of Hong Kong, New Territories, Hong Kong, 999077 China; 8 Present Address: Department of Chemistry Education, Graduate Department of Chemical Materials, Institute for Plastic Information and Energy Materials, Sustainable Utilization of Photovoltaic Energy Research Center/Engineering Research Center, Pusan National University, Busan, 46241 Republic of Korea

**Keywords:** Solar cells, Materials for energy and catalysis

## Abstract

The non-fullerene acceptors (NFAs) employed in state-of-art organic photovoltaics (OPVs) often exhibit strong quadrupole moments which can strongly impact on material energetics. Herein, we show that changing the orientation of Y6, a prototypical NFA, from face-on to more edge-on by using different processing solvents causes a significant energetic shift of up to 210 meV. The impact of this energetic shift on OPV performance is investigated in both bilayer and bulk-heterojunction (BHJ) devices with PM6 polymer donor. The device electronic bandgap and the rate of non-geminate recombination are found to depend on the Y6 orientation in both bilayer and BHJ devices, attributed to the quadrupole moment-induced band bending. Analogous energetic shifts are also observed in other common polymer/NFA blends, which correlates well with NFA quadrupole moments. This work demonstrates the key impact of NFA quadruple moments and molecular orientation on material energetics and thereby on the efficiency of high-performance OPVs.

## Abstract (web summary)

Non-fullerene acceptors with large quadrupole moments strongly affect thin-film energetics. Here, the authors show a significant energetic shift (>200 meV) of Y6 upon changing its orientation from face-on to more edge-on using different solvents and its critical impact on organic photovoltaics.

## Introduction

Organic photovoltaics (OPVs) are attracting significant attention due to their potential to be lightweight, flexible, non-toxic, and compatible with large-scale manufacturing. Since 2015, the development of the new small molecule-based non-fullerene acceptors (NFAs)^1^ has enabled OPVs to show remarkable improvements in both power conversion efficiency (PCE) and device stability^2^. In recent studies, single-junction and tandem OPVs have been reported to achieve PCEs of over 19 and 20%, respectively^3,4^. However, such OPVs are still suffering from significant open-circuit voltage (*V*_OC_) losses, which limit the PCE of single-junction OPVs compared to the reported theoretical limit of 20–25%^5^.

The state-of-art organic photovoltaics (OPVs) utilize the bulk-heterojunction (BHJ) structure where electron donor polymer and electron acceptor NFA materials are blended together to form a single photoactive layer. For such BHJ devices, it is well known that the energetics at the donor-acceptor heterointerface (herein denoted as heterointerface) play a vital role in determining both the efficiency of free charge generation^6,7^ and the magnitude of the *V*_OC_ loss^8–10^. In most of such devices, hole transfer from the lower bandgap acceptor to the donor is the key step for free charge generation, and the efficiency of this process is determined by the offset between the highest occupied molecular orbital (HOMO) levels of donor and acceptor. Meanwhile, this HOMO offset also affects the effective bandgap (the energy gap between donor HOMO and acceptor LUMO, where LUMO stands for the lowest unoccupied molecular orbital), which sets the upper limit for the device *V*_OC_. Therefore, accurate determination and precise control of the HOMO offset are crucial for OPVs to approach their theoretical performance limit.

Besides their intrinsic molecular properties, other factors such as intermolecular couplings induced by induction and electrostatic interactions also play important roles in determining the energy levels of organic thin films. The induction effect in thin films typically aligns molecules in close proximity and therefore governs the packing structure and the thin film morphology^11^. Electrostatic effects, on the other hand, can provide a background electric field which results in energy level shifts in the thin films^12^. Several studies have suggested that dipole moments, either intrinsic molecular dipoles^13–15^ or interfacial dipoles formed by ground state charge transfer between donor and acceptor molecules^16^ can result in significant energetic shifts in organic thin films. However, others have argued that as dipole moments are usually aligned and canceled in organic thin films due to their symmetric molecular structures and particular packing structures, the quadrupole moment determines the dominant electrostatic interaction and hence the energy level shifts^7,17,18^. The effect of quadrupole moments on the electrostatic effect has been previously studied in sublimed organic small molecule thin films, including zinc(II)-phthalocyanine, sexithiophene (α−6T)^7^, pentacene^19^ and their derivatives. These studies showed a consistent shift of the energetics correlated with their quadrupole moments and packing orientations. Since conjugated molecules are closely packed along their π-π stacking direction, the electrostatic effect induced by the quadrupole moment is usually dominated by its component along this π-π stacking direction (Q_π_)^7^. Due to physical confinement of a thin film, different molecular orientations (e.g., face-on and edge-on) with respect to the substrate will result in different convergence lengths leading to different energy levels^20^. For example, Dong et al. showed that there is a 0.4 eV energetic shift between face-on and edge-on evaporated α−6T thin films, which provides a sufficient driving force to split excitons and generate free charges in a single component α−6T OPV device^21^.

Compared to sublimed small molecule films where the molecular orientation control is relatively easy, there has been no report, to the best of our knowledge, demonstrating the orientation control of solution-processed NFA molecules leading to an energetic shift large enough to impact exciton separation for free charge generation. Most high-performing NFAs for OPVs such as ITIC, IDTBR and Y6 have a planar backbone structure with alternating acceptor and donor moieties (typically in A-D-A or A-D-A’-D-A structures). Such symmetric molecular structures and particular packing structures in thin films can induce a negligible net dipole moment in NFA thin films^22^. Instead, such A-D-A or A-D-A’-D-A structures can induce an enhanced inhomogeneous charge distribution along the conjugated backbone of the molecule due to incorporation of strong A and D units, resulting in a large Q_π_^23^. Recent studies of NFA-based BHJ OPVs attributed their excellent device performances to the Q_π_ of employed NFAs, including Y6^23^, ITIC^24^, and O-IDTBCN^25^. In those studies, a band bending at heterointerface is proposed, arising from the molecular quadrupole moments of NFAs and the concentration gradient of donor and acceptor species between interfacial and bulk regions^26^. However, no direct measurements of such quadrupole moment-induced energetic shifts and their impact on OPV performance have been reported. Moreover, the previous studies mainly focused on controlling the Q_π_ of NFAs in BHJ devices by changing NFA molecular structures. As the impact of Q_π_ is also expected to be highly dependent on NFA orientation, a detailed study on the molecular orientation-dependent energetic shift in solution-processed NFAs and its impact on OPV performance is still needed, which is the main aim of this work.

In this work, we select Y6 molecule, one of the high-performing solution-processed NFAs for OPV devices and control its molecular orientation in thin films from face-on to more edge-on by using different solvents for spin-coating. First, we determine the impact of Y6 orientation on its energetics using Ambient photoemission spectroscopy (APS)^27^, which is more relevant for samples and devices fabricated under the ambient condition compared to commonly used Ultraviolet photoelectron spectroscopy (UPS)^16,28^. The HOMO level is observed to deepen by 210 meV in neat films as Y6 molecules switch from face-on to more edge-on orientation, which is well correlated with its large positive Q_π_ value, calculated as 192 ea_0_^2^, affecting its electrostatic potential. Such an orientation-dependent energy level shift has a direct impact on the energetics at the polymer donor and NFA acceptor heterointerfaces. Y6/PM6 bilayer structures were fabricated by transferring the PM6 film on top of the differently oriented Y6 films^29,30^. The change in Y6 orientation from more edge-on to face-on also impacts the energy levels of PM6, pulling its HOMO down by 100 meV near the heterointerface. Second, we directly measure the effect of such Y6 orientation-dependent energetic shift on the *V*_OC_ of OPVs by employing differential capacitance and transient photovoltage analyses. The face-on Y6 device exhibits a 0.22 eV increase in the electronic bandgap, in good agreement with the HOMO level offset measured by APS. However, this increase is partially offset by a 50-fold decrease in charge carrier lifetime, attributed to a loss of quadrupole-induced band bending at the Y6/PM6 heterointerface, leading to a 0.11 V net loss in calculated *V*_OC_, in excellent agreement with the directly measured *V*_OC_ shift under 1 sun. The larger quadrupole-induced band bending observed in the more edge-on device is found to assist better exciton and charge separation, resulting in an increased internal quantum efficiency for photocurrent generation following Y6 excitation. Third, we further show that such molecular-orientation-dependent energetic shifts are present not only in the planar structures but also in BHJ D:A blend systems. In PM6:Y6 BHJ devices, we find that the Q_π_ of Y6 also alters the energetic offset in blend films, leading to similar trends in *V*_OC_ (0.05 V difference) and charge generation. These results indicate that even in BHJ devices, the strong Q_π_-induced electrostatic potential effect still exists affecting the D/A energetics and hence directly changing *V*_OC_. Finally, we extend our study to other commonly used polymer donor:NFA BHJ systems. A clear correlation between the magnitude of the polymer donor energetic shift and the magnitude NFA Q_π_ is found. Our results demonstrate the key impact of NFA quadruple moments and molecular orientation on material energetics and thereby on the efficiency of high-performance OPVs.

## Results

### Orientation-dependent energetic shift in neat films

**Fig. 1:**
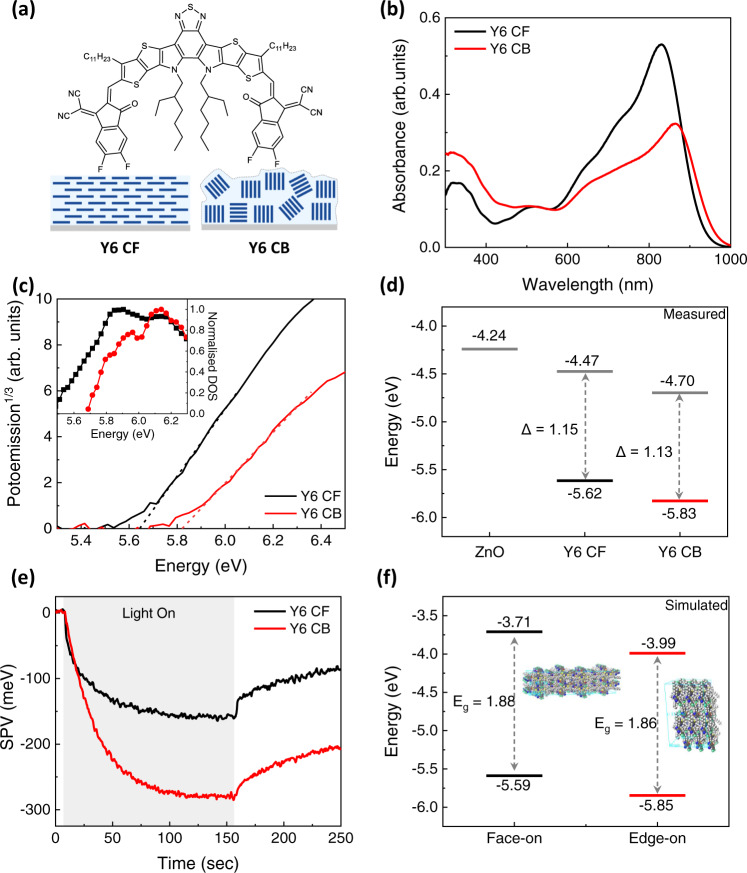
Impact of Y6 orientation on neat film energetics and charge generation. **a** The molecular structure of Y6 and the schematic of packing structure of Y6 molecules in the film processed by chloroform (CF) and chlorobenzene (CB). **b** Absorption spectra and **c** ambient photoemission spectroscopy (APS) spectra of Y6 CF and Y6 CB. The inset shows the normalized density of states. **d** HOMO and Fermi level (E_F_) of ZnO, Y6 CF and Y6 CB with constant energy differences, Δ = | HOMO-E_F_ | of Y6 with different preparation methods highlighted. **e** Surface photovoltage (SPV) measurements of Y6 CF and Y6 CB on ITO/ZnO substrates. **f** HOMO and LUMO levels of Y6 thin films with face-on and edge-on orientation with respect to substrate simulated using molecular dynamics and DFT, taking into account the electrostatic potential induced by partial charges on surrounding Y6 molecules. The similar bandgaps E_g_ in both cases are highlighted.

The molecular orientation of Y6 (molecular structure in Fig. 1a) in neat films can be controlled by choosing solvents with different boiling points, such as chloroform (CF) and chlorobenzene (CB). Grazing incidence wide-angle X-ray scattering (GIWAXS) measurements show that the (010) π-π stacking peak of Y6 has a wider polar angle distribution in neat Y6 film deposited from CB (denoted as Y6 CB) compared to that deposited from CF (denoted as Y6 CF), indicating more edge-on orientations in Y6 CB film (Supplementary Fig. 1). Atomic force microscopy (AFM) reveals that Y6 CB has a much higher surface roughness (*R*_q_ = 26.4 nm) than Y6 CF (*R*_q_ = 1.26 nm) due to the mixed orientations and strong aggregation in the Y6 CB film (Supplementary Fig. 2a, b). These results indicate that Y6 CF adapts a meta-stable face-on orientation, while Y6 CB has more edge-on mixed orientations with large crystallites, consistent with previously reported morphological data^31–33^. Overall schematics of thin film morphologies are depicted in Fig. 1a. The absorbance of Y6 CB is much lower than that of Y6 CF, despite their similar thickness of around 40 nm (Fig. 1b). This is attributed to more mixed orientations of Y6 CB reducing the in-plane absorption. The optical bandgaps of Y6 CF and CB are determined to be 1.41 and 1.38 eV, respectively, from the intersection of their normalized absorbance and PL spectra (Supplementary Fig. 3)^34^. The smaller bandgap in CB can be attributed to the formation of stronger aggregation that enhances the carrier delocalization, similar to what observed by Zhu et al.^31^. The resonant Raman spectra also show much lower intensity for Y6 CB than for Y6 CF with intact peak shape, position, and relative intensity (Supplementary Fig. 4), indicating solvent choice mainly influences the molecular orientation and aggregation while keeping the molecular conformation unchanged.

Turning to the impact of molecular orientation on energetics, the HOMO and Fermi levels (E_F_) of Y6 CF and Y6 CB films were measured by APS and Kelvin probe^21,22,35,36^. The HOMO level of as-cast Y6 CB is observed at −5.83 eV, which is 210 meV deeper than that of Y6 CF (−5.62 eV) (Fig. 1c). After thermal annealing at 100 °C, this large HOMO level difference is maintained with an additional deepening of HOMO to −5.67 and −5.86 eV for Y6 CF and Y6 CB, respectively (Supplementary Fig. 5a). Nonetheless, the E_F_-HOMO gap remains nearly constant between Y6 CF and CB for both as-cast and annealed films (Fig. 1d and Supplementary Fig. 5b). Additionally, the similar optical bandgaps of Y6 CF and CB (Supplementary Fig. 3) suggests that the LUMO is also shifted in a similar way. The simultaneous shift of HOMO, E_F_ and LUMO indicates that the large energetic shift up to 210 meV mainly originates from electrostatic effects induced by different Y6 orientations^7,11,12,17^. Here we emphasize that due to the relatively large probing depth of APS (5–10 nm)^27^, the measured HOMO levels should mainly reflect the bulk properties.

To further investigate the effect of Y6 orientation on photoinduced charge generation/extraction, we measured the surface photovoltage (SPV) of differently oriented Y6 samples (ITO/ZnO/Y6 CF or Y6 CB). As shown in Fig. 1e, upon illumination, an additional negative 130 mV SPV response was measured for Y6 CB compared to Y6 CF. The negative SPV indicates the redistribution of photoinduced charge carriers: accumulation of holes at the Y6 top surface after electrons being extracted via the bottom ZnO electron transporting layer^37,38^. Much higher negative SPV in Y6 CB indicates more efficient photoinduced charge generation. This possibly arises from the presence of large domains with more mixed orientations (both face-on and edge-on) in Y6 CB (Fig. 1a), as the energetic shift at differently oriented grain boundaries may provide a driving force for exciton dissociation and free charge generation in neat film, similar to the α−6T case as reported by Dong et al.^21^. We note that the dissociation of Y6 excitons at the Y6-ZnO interface may be another source of free charge generation^39^. However, this cannot explain the stronger SPV response measured in Y6 CB as its much deeper LUMO would make electron transfer to ZnO energetically unfavorable^40^. To exclude this possibility, we also measured exciton lifetimes in Y6 CF and CB deposited directly on top of quartz using time-correlated single photon counting (TCSPC). As shown in Supplementary Fig. 6, the shorter lifetime in Y6 CB (τ = 590 ps) compared to Y6 CF (τ = 890 ps) suggests faster exciton dissociation in Y6 CB and the subsequent free charge generation results in the larger SPV signal we measured. Our observation is in line with the recently published works on charge (or intermediate state) photogeneration in neat Y6 films^41,42^, and shows the importance of molecular orientation and packing in thin films for free charge generation due to the large molecular quadrupole moments of NFAs.

To confirm that such large electrostatic energetic shifts observed with different molecular orientations originate from molecular quadrupole moment, the Q_π_ values of well-known NFAs and small molecular donors were simulated by density functional theory (DFT). High-efficiency NFAs commonly possess an A-D-A or A-D-A’-D-A structure which produces a strong push-pull effect across the conjugated backbone. The strong electron-withdrawing moieties on both sides leave the central donating moiety electron deficient as shown in the electrostatic potential mapping (Supplementary Fig. 7a), resulting in a much larger positive Q_π_ in NFAs than the small molecular donors such as a-6T reported previously (Supplementary Fig. 7b)^7,22,43^. In particular, Y6 shows a large positive Q_π_ of 192 ea_0_^2^, which can account for the large energetic shift with different molecular orientations as observed in Y6 CF and Y6 CB (40 nm). To gain a deeper understanding of the quadrupole effect, molecular dynamics (MD) together with DFT (b3lyp/6-31 G**) simulations are applied, and the results are shown in Fig. 1f. When the charge-quadrupole electrostatic interactions are taken into account, a clear shift in both HOMO (260 meV) and LUMO (280 meV) levels is seen between face-on and edge-on oriented Y6 films, keeping the bandgap nearly unchanged (1.88 vs.1.86 eV), which agrees well with the energetics (by APS) and optical (by UV-Vis absorption) measurements. A detailed inspection of the MD-simulated packing structures with the molecular partial charges visualized (see Supplementary Fig. 8) allows us to explain the large energetic shift: the edge-on oriented Y6 molecules form more ordered eclipsed stacking structure compared to face-on oriented Y6 molecules with alternate stacking structure. Since quadrupole potential has an inverse-cube distance dependence, this will result in a stronger superposition of the Y6 Q_π_ fields in the edge-on case, hence leading to a stronger downward energetic shift. The simulation results also align well with our GIWAXS data (Supplementary Fig. 1), where Y6 CF has a looser packing along the π-π stacking direction (d_010_ = 3.63 Å) compared to Y6 CB (d_010_ = 3.59 Å). Additionally, for Y6 CF with face-on orientation, other components of quadrupole moment (with opposite signs) will compete with Q_π_, while energy levels in Y6 CB with a more edge-on orientation is more dominated by Q_π_, resulting in a much larger downwards energetic shift. As Y6 and its derivatives have been observed to show stronger intermolecular electronic couplings compared to other NFAs^44^, the potential band dispersion effect (e.g., electronic coupling) in Y6 CF and CB may be another reason accounting for the energetic shift. To exclude this possibility, further DFT calculations are carried out on dimer structures randomly extracted from the MD-simulated face-on and edge-on packings (10 each) without any electrostatic interaction. For each dimer pair, the HOMO/LUMO energy levels and respective electronic coupling values are calculated (see Supplementary Fig. 9a, b) and the average values for face-on and edge-on packings are shown in Supplementary Fig. 9c. Despite the edge-on packing showing a larger electronic coupling value compared to the face-on packing, the average energy of HOMO and LUMO are very similar in both packing structures, confirming that the energetic shift measured indeed originates from charge-quadrupole interaction. At the boundary of the crystals (i.e., film/air interface), this electrostatic energetic shift will diminish, causing an upward band bending of the Y6 energy levels originating from its positive Q_π_ value. With a much stronger Q_π_-induced energetic shift in the bulk, Y6 CB is therefore expected to have a larger upward band bending near the air interface compared to Y6 CF. Overall, the band diagrams of Y6 neat films affected by the positive Q_π_ depending on molecular orientations: face-on and edge-on are drawn in Supplementary Fig. 7c. Such larger interfacial band bending can also lead to larger negative SPV in Y6 CB film as measured (Fig. 1e), indicating more efficient photoinduced charge generation. Overall, these results indicate that the up to 210 meV energetic shift in solution-processed neat Y6 films is achievable with a simple molecular orientation change and this change can be attributed to the large positive Q_π_ value of Y6 generating strong electrostatic interactions between the molecules.

### Orientation-dependent energetic shift and charge carrier dynamics in bilayer devices

**Fig. 2:**
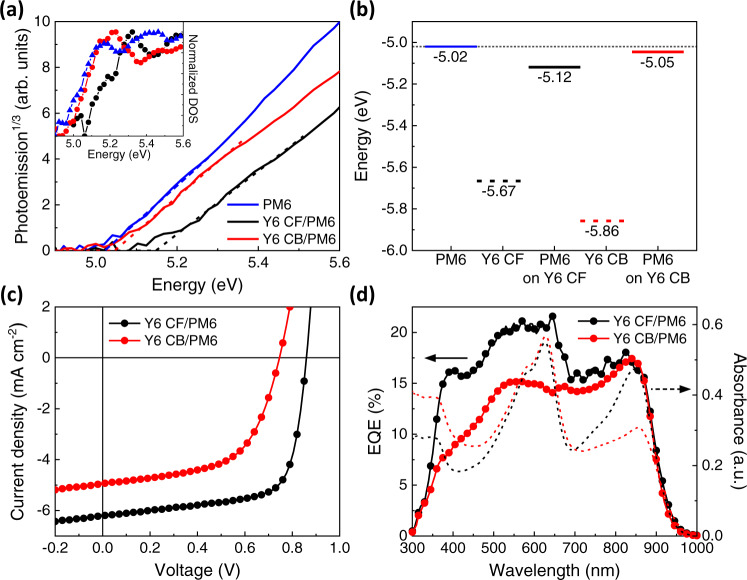
Impact of Y6 orientation on PM6 energetics in bilayer film and device performance. **a** APS spectra of 5 nm PM6 layer on top of Y6 CF and Y6 CB compared to neat PM6 layer on the ITO/ZnO substrate. The inset is the normalized density of states. **b** HOMO levels of neat PM6, Y6 CF/PM6, and Y6 CB/PM6 films. **c** *J*-*V* characteristics and **d** external quantum efficiency (EQE) spectra of Y6/PM6 bilayer devices overlaid with the absorbance of corresponding films.

Next, we investigate the influence of Y6 molecular orientations on energetics of PM6 layer deposited on top of the Y6 film. Bilayer samples were fabricated by spin coating Y6 CF or Y6 CB on ITO/ZnO substrates and then depositing PM6 on top of it using a film transfer method^29,30^. A well-defined planar heterojunction is formed with an intact Y6 layer underneath, and the thickness of PM6 layer controlled by the concentration of PM6 solution. As shown in the APS spectra (Fig. 2a), the HOMO level of neat PM6 is −5.02 eV, which is more than 0.6 eV shallower than the HOMO level of Y6 CF and Y6 CB (Fig. 1c, d), allowing us to confidently assign the photoemission onset of bilayer films to the HOMO levels of PM6 despite the large probing depth of APS. Noticeably, we found that the HOMO of PM6 deepens by 100 meV at the Y6/PM6 interface when PM6 was deposited on top of Y6 CF. This difference, interestingly, becomes minor when the thickness of PM6 layer was increased to above 5 nm (Supplementary Fig. 10), revealing the short-range interfacial PM6 band bending induced by Y6 quadrupole moment. Note that due to the low Q_π_ value of PM6 (Supplementary Fig. 7b), its influence is negligible compared to Y6. Similarly as explained earlier, moving from the bulk of the underlying Y6 film to the PM6:Y6 heterointerface, the impact of Y6 positive Q_π_ will diminish leading to upward band bending of Y6 energy levels, concomitantly pulling down the polymer energy levels (see the schematics in Supplementary Fig. 11)^28,46^. The HOMO level of PM6 is less affected by Y6 CB possibly due to the more mixed nature of Y6 CB orientations, generating rougher heterointerfaces with PM6 and lowering the net Y6 Q_π_ effect^45^. This explains the 100 meV HOMO shift for the 5 nm PM6 thin film on top of the Y6 CF but only a minor shift on top of the Y6 CB. Similar band bending at heterointerfaces has also been predicted by Markina et al.^26^. Overall, as summarized in Fig. 2b, the bulk HOMO levels of annealed Y6 CF and CB differs by 190 meV due to their different molecular orientations inducing different extent of charge-quadrupole interaction. At the heterointerface, there is an additional 100 meV deepening of PM6 HOMO induced by Y6 Q_π_ when PM6 is deposited on top of Y6 CF. Note that we used energy levels of annealed Y6 CF and CB films here to align with the experimental conditions for bilayer device preparation (see Method).

A large vacuum level shift has been reported at the NFA/polymer heterointerfaces, where organic layers are fabricated by a mono-layer-by-layer deposition technique^16^. It is attributed to a possible formation of the ground-state interfacial dipoles (i.e., charge transfer states) between donor polymer and NFA, which induces the possible energetic offset for charge generation. A particular intermolecular arrangement between NFA and polymer, i.e., strong electron-withdrawing units in NFA facing strong electron-donating units in polymer, can be expected to assist the formation of the ground-state charge transfer states. However, we did not observe a significant vacuum level shift across the heterointerface when the contact potential difference was measured in ambient conditions before and after depositing a 5 nm PM6 layer on top of Y6 CF and Y6 CB films (Supplementary Fig. 12). This indicates that negligible interfacial dipoles are formed at these Y6/PM6 heterointerfaces and the quadrupole-moment induced electrostatic potential shift arising from different Y6 orientations is still the dominant factor determining interfacial energetics measured here. Note that the A-D-A or A-D-A’-D-A type NFAs show very small intrinsic dipole moments, e.g., ITIC (0.45 Debye)^22^, IDTBR (0 Debye)^22^ and Y6 (0.87 Debye)^46^, which unlikely impacts the interfacial energetics.

To investigate the Q_π_ effect of Y6 on the photovoltaic device performance, bilayer OPV devices were fabricated with an architecture of ITO/ZnO/Y6 (CF or CB)/PM6/MoO_3_/Ag. As shown in current density-voltage (*J*-*V*) curves (Fig. 2c), the different molecular orientations of Y6 have a significant impact on device performance. The Y6 CF device (denoted as CF device) shows an improvement of all photovoltaic parameters compared to the device with Y6 CB (denoted as CB device) – *J*_SC_ (6.21 *vs*. 4.95 mA cm^−^^2^), *V*_OC_ (0.86 *vs*. 0.75 V) and FF (0.699 *vs*. 0.578) – thus leading to a higher PCE (3.73% *vs*. 2.13%).

Figure 2d overlays EQE and optical absorbance spectra for Y6 CF/PM6 and Y6 CB/PM6 bilayer devices, respectively. The higher *J*_SC_ in the CF device can be attributed to higher external quantum efficiency (EQE) between 350 nm and 650 nm (Fig. 2d), in the region of PM6 absorption. Interestingly, both the CF and CB devices have similar absorbance in this region. This indicates a higher internal quantum efficiency (IQE) for the CF device, which could be due to more efficient Förster resonance energy transfer (FRET) or electron transfer from PM6 excitons to Y6. We note the absorbance of Y6 CF has more spectral overlap with PM6 PL, and also, the face-on oriented Y6 CF has a better-aligned transition dipole which could further improve the energy transfer process (see Supplementary Fig. 13a, b)^47,48^. Ultrafast (sub-ps) electron transfer in PM6:Y6 has been observed in several previous studies mainly due to the large LUMO offset in this system^35,42^, so the different LUMO offsets in the CF and CB devices (1.03 and 1.32 eV, respectively, estimated from HOMO and optical bandgap) should not influence the rate of electron transfer. Instead, the more systematic orientation in the CF device would maximize the wavefunction overlap of donor and acceptor molecules at the heterointerface, accelerating the electron transfer process. On the other hand, in the wavelength region above 650 nm, the EQE of both devices are comparable despite the lower absorbance of CB device, suggesting higher IQE for the CB device following Y6 photoexcitation. Both devices show similar Y6 PL quenching by the PM6 top layer (see Supplementary Fig. 13c, d), which suggests a similar exciton dissociation efficiency at the heterointerface. Therefore, the more efficient exciton-to-electron conversion for the CB device in this Y6 spectral region may be attributed to the following reasons. Firstly, our ultrafast transient absorption data indicates suppressed recombination losses in the CB device (Supplementary Figs. 14 and 15), which could result from stronger quadrupole-induced band bending at this heterointerface, as we will discuss below^24–26^. The shorter carrier lifetime in the CF device results in a stronger nongeminate recombination loss under the short-circuit condition as reflected from the intensity-dependent *J*_SC_ measurements (see Supplementary Fig. 16a) where exponent *α* obtained from the power-law fitting is smaller for the CF device (0.97) compared to the CB device (1.00). Additionally, there may also be free charge generation within Y6 CB layer as evident from our neat film SPV results discussed earlier^21,41,42^. This will help dissociate Y6 excitons that are not able to diffuse to Y6/PM6 heterointerface, further increasing the efficiency of exciton harvesting.

**Fig. 3:**
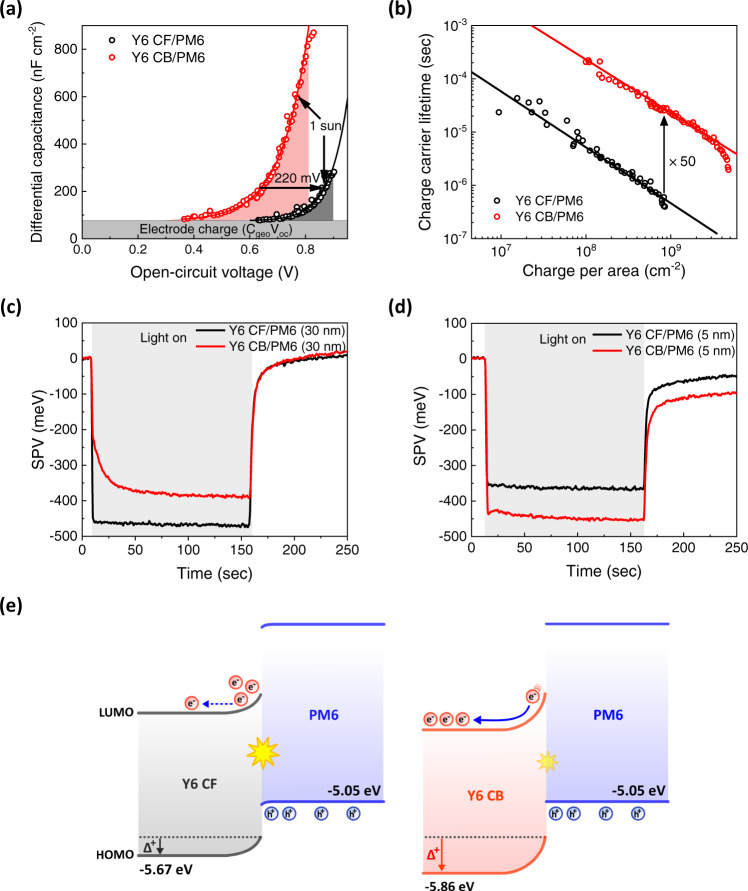
Analysis of charge extraction and recombination mechanisms. **a** Differential capacitance *vs*. open-circuit voltage and **b** Charge carrier lifetime determined from TPV *vs*. the spatially averaged charge carrier density determined from the differential charging method of the Y6 CF/PM6 and the Y6 CB/PM6 device. The difference in charge carriers stored in the active layer under 1 sun illumination and the shift in the electronic bandgap are highlighted in (**a**). SPV measurements of **c** 30 nm PM6 on Y6 CF and Y6 CB, and **d** 5 nm PM6 on Y6 CF and Y6 CB. **e** A schematic of energy levels in bilayers to illustrate the effect of the quadrupole moment-induced band bending on interfacial recombination in bilayer devices. Δ^+^ indicates the interfacial band bending by the positive quadrupole moment.

To understand the difference in *V*_OC_, we firstly probe the electronic bandgaps in both CF and CB bilayer devices via differential capacitance measurements as a function of light intensity, which describes the evolution of the quasi-fermi level splitting (QFLS) as charge carriers build up within the photoactive layer^49,50^. Figure 3a shows the differential capacitance of the devices plotted against the *V*_OC_, where *V*_OC_ is used as an approximation of the QFLS within the active layer. After subtracting the constant electrode capacitance, both devices show the expected exponential increase of the active layer chemical capacitance (see fitted lines in Fig. 3a). Two features become apparent: firstly, the capacitance and hence the charge stored in the active layer (illustrated by the shaded area under the curve) is significantly higher in the CB device compared to the CF device at matched light intensities (e.g., 1 sun as indicated). Secondly, the built-up of charges in the CF device is shifted towards higher values of *V*_OC,_ signifying an apparent shift of the electronic bandgap by about 220 meV (determined at 1 sun). We attributed this difference in the electronic bandgaps mainly to the 190 meV shift of bulk HOMO levels between annealed Y6 CF and CB measured by APS (Fig. 2b). The agreement in results obtained from both methods emphasizes the important effect of orientation-dependent charge-quadrupole interaction, which directly alters the electronic bandgap in OPV devices. At the D:A heterointerface, the quadrupole effect will decay, resulting in an upward band bending of Y6 energy levels similar to the scenario at the film/air interface, although we cannot directly measure the exact interfacial CT state energy from the electroluminescence spectra due to the dominance of Y6 emission (Supplementary Fig. 17)^51,52^. The deeper bulk HOMO of Y6 CB will therefore induce a larger band bending at the heterointerface, as depicted in Fig. 3e, which significantly influences carrier distribution and recombination in bilayer devices, as discussed below.

Transient photovoltage (TPV) was conducted to assess the recombination kinetics of the charge carriers across the active layer at open-circuit condition, as shown in Fig. 3b. Comparing the charge carrier lifetime at similar charge carrier densities (e.g., 1 sun of the CF device), it becomes apparent that the CB device exhibits an approximately 50-fold increase in the charge carrier lifetime, which we assign to the change in the charge carrier distribution across the bilayer device. While in BHJ devices charge carriers are often thought to be uniformly distributed across a single effective medium when assessing TPV kinetics, this clearly is not the case for the bilayer devices, as studied here. Rather, we highlight that the quadrupole effects discussed above will provide greater band bending in the CB device which lowers the electronic bandgap but also drives charges away from the Y6/PM6 heterointerface even if the interfacial energetics are the same. We propose that this quadrupole-induced band bending is the primary origin of the slower non-geminate (bimolecular) recombination kinetics we observe herein for the CB device as carriers far away from the heterointerface are at least partially screened from recombination. Apart from interfacial carrier concentration, the magnitude of non-geminate recombination in bilayer devices also depends on the interfacial area. However, this can be ruled out as the origin of the slower recombination kinetics of the CB devices, as this device with a rougher Y6 layer (see AFM images in Supplementary Fig. 2a, b), and so a larger Y6/PM6 heterointerface area, would accelerate non-geminate recombination in this device^8,53^, which is opposite to our experimental observation. Additionally, the extent of charge carrier delocalization at D:A heterointerface has also been suggested to determine the rate of non-radiative recombination^54,55^. However, only carrier delocalization in the face-on system can effectively suppress non-radiative recombination by moving the centroid of the carrier wave function away from the heterointerface, which is again opposite to our observation. Rather our differential charging and TPV analysis highlight the impact of quadrupole-induced interfacial band bending on charge carrier distribution and non-geminate recombination losses in these devices.

Using the measured charge carrier densities and lifetimes together we can quantify the kinetic contribution to the *V*_OC_ difference between the two devices using the Eq. (1)^56^:

1 $$\varDelta {V}_{{OC}}^{{kinetic}}=-\frac{{n}_{{id}}{{{{{\rm{kT}}}}}}}{q}{{{{{\rm{ln}}}}}}\left(\frac{{J}_{{BI}}^{{CF}}}{{J}_{{BI}}^{{CB}}}\cdot \frac{{J}_{{gen}}^{{CB}}}{{J}_{{gen}}^{{CF}}}\right)$$

where *n*_id_ is the ideality factor measured to be close to 1 for the CF device (see Supplementary Fig. 16b), kT is the thermal energy, *q* is the elementary charge, *J*_BI_ and *J*_gen_ denote the non-geminate loss current $\left(\frac{{J}_{{BI}}^{{CB}}}{{J}_{{BI}}^{{CF}}}=\frac{1}{50}\right)$ and generation current (approximated as *J*_SC_), respectively. We calculate an extra kinetic loss of *V*_OC_ of about 110 mV for the CF device compared to CB, explaining why the 220 meV increase in electronic bandgap only results in 110 mV increase in *V*_OC_ for CF device. To further underline the validity of our analysis, the reconstruction of the light intensity-dependent *V*_OC_ from our experimentally fitted charge carrier density and lifetime data shows excellent agreement with the experimentally measured *V*_OC_ values (Supplementary Fig. 16b).

To explore further the impact of quadrupole-induced interfacial band bending on charge accumulation and recombination, we perform SPV measurements on bilayer samples (ITO/ZnO/Y6/PM6) with different PM6 layer thicknesses^37,38,57^. As shown in Fig. 3c, when the thickness of PM6 layer is 30 nm, which is the same as that in bilayer devices, the SPV response of the CF sample (−470 mV) is larger than that of the CB sample (−390 mV), in qualitative agreement with the device *V*_OC_^58^. However, as the thickness of PM6 layer is reduced to 5 nm (Fig. 3d), the SPV response of the CF sample (−369 mV) becomes smaller than that of the CB sample (−453 mV). The smaller SPV magnitude of the CF sample at lower PM6 layer thickness is consistent with the strong recombination loss originating from a higher concentration of electrons accumulated at the heterointerface. On the contrary, the CB device with much slower interfacial recombination shows an increase in the SPV signal with reduced PM6 thickness, suggesting a significantly reduced accumulation of electrons at the heterointerface due to stronger interfacial band bending, as illustrated in Fig. 3e. This explains the higher overall charge carrier density stored in the CB device as measured by differential capacitance at the same light intensity. The increase in SPV response with decreasing PM6 layer thickness in the CB sample also aligns with the lower EQE of the CB bilayer device measured in the spectral region of PM6 absorption (Fig. 2d). Only the PM6 excitons generated near the heterointerface can be harvested. As a result, decreasing the PM6 layer thickness in the CB sample confines the same amount of photogenerated holes into a thinner layer, leading to an increase in the SPV response^37^.

In summary, due to the large positive Q_π_ of Y6, the orientations of Y6 have a strong impact on both the bulk energetics and interfacial band bending, which governs the electronic bandgap and the interfacial recombination rate, both of which decide the overall *V*_OC_ loss in this bilayer system.

### Orientation-dependent energetic shift and its impact on BHJ device performance

**Fig. 4:**
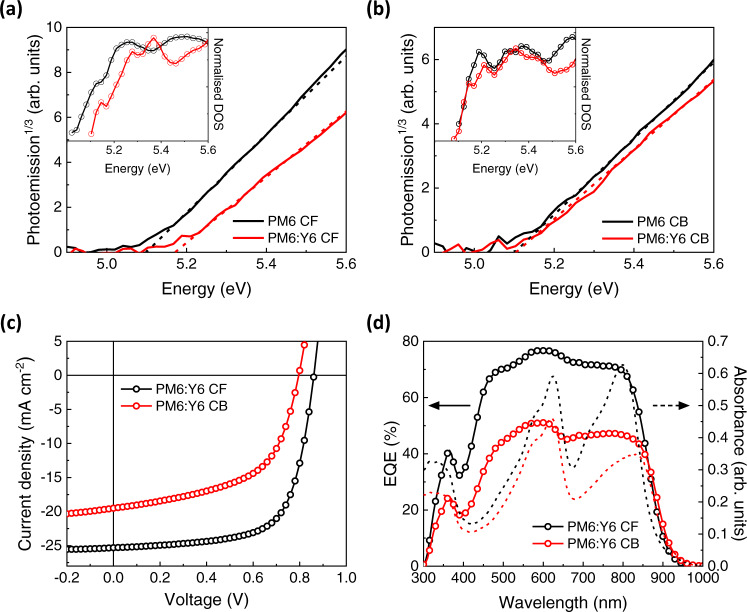
Influence of processing solvents on energetics and performances of PM6:Y6 BHJ devices. APS spectra of neat PM6 and PM6:Y6 blend films processed from **(a)** CF and **(b)** CB. DOS distributions are shown in the inset. **c** *J*-*V* characteristics and **d** EQE spectra of PM6:Y6 BHJ devices overlaid with the absorbance of corresponding films.

To investigate if such quadrupole-induced electrostatic effect also exists when Y6 is blended with polymer donors, we further characterize PM6:Y6 BHJs processed from CF or CB. Learning from previous studies and our AFM images (Supplementary Fig. 2e, f), it appears Y6 inside the CB BHJ maintains a mixed orientation with large phase separation and rougher surface features while both PM6 and Y6 inside CF BHJ show a preferential face-on orientation with smaller phase separation and a smoother surface^31,33,59^. As shown in Fig. 4a, upon BHJ formation with Y6, an 87 meV deepening of the PM6 HOMO is observed for CF, while only minor changes occur for CB (< 10 meV, Fig. 4b), consistent with the changes in the DOS only observed for CF (see insets of Fig. 4a, b). On the other hand, the optical bandgap of PM6 remains similar in both CF and CB BHJs as shown in the absorption spectra normalized with respect to PM6 (Supplementary Fig. 18). This confirms that the larger energetic shift for the CF BHJ is due to the stronger charge-quadrupole interactions between PM6 and Y6, such that the HOMO of PM6 is pulled down by the positive Q_π_ of Y6, like the bilayer energetics mentioned in the previous section. We attributed this to the better-aligned PM6:Y6 orientation and smaller phase separation, maximizing the superposition of their different Q_π_ fields^45^. As a result, we expected a reduction in the HOMO offset and an increase in the electronic bandgap in the CF BHJ device. *J*-*V* characteristics of the corresponding BHJ devices (ITO/ZnO/PM6:Y6/MoO_3_/Ag) are shown in Fig. 4c. The CF BHJ yields superior *J*_SC_ (25.5 *vs*. 20.2 mA cm^−2^) and *V*_OC_ (0.86 *vs*. 0.80 V), leading to an overall higher PCE. The face-on orientation results in higher EQE over the whole spectrum for the CF BHJ, which is mainly due to the larger absorbance (Fig. 4d). Additionally, the larger domain size (see Supplementary Fig 2e and f) in the CB BHJ may impede efficient exciton harvesting, and the slower charge extraction (Supplementary Fig. 19) results in additional non-geminate recombination loss (Supplementary Fig. 20), both of which can account for the lower EQE measured. The higher *V*_OC_ in CF BHJ can be explained by its higher electronic bandgap due to stronger charge-quadrupole interaction (Fig. 4a, b), which is also confirmed by differential capacitance measurement (Supplementary Fig. 21a). The increase in the electronic bandgap of the CF BHJ device is offset by a lower charge carrier lifetime as measured by TPV (Supplementary Fig. 21b), analogous to the bilayer device. Overall, the consistent results obtained from both bilayer and BHJ devices processed from CF and CB point towards a unified understanding of the quadrupole effect at D/A heterointerfaces, in this case predominantly controlled by the NFA molecular quadrupole moment, orientation, packing and phase separation. The strong quadrupole effect observed in the bilayer system is also applicable to the BHJ system, albeit less pronounced as the mixing of donor and acceptor in BHJ films disturbs the superposition of molecular quadrupole moments.

**Fig. 5:**
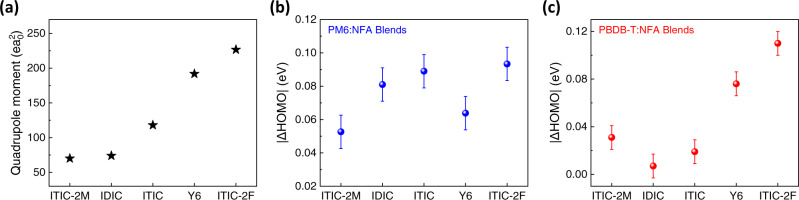
Quadrupole effect on HOMO level shift (ΔHOMO) of polymer donors upon blending with different NFAs in CF. **a** Quadrupole moments of NFAs and the magnitude of ΔHOMO of **b** PM6 and **c** PBDB-T increase as the DFT simulated Q_π_ of NFAs increases. (Error bars are the intrinsic machine error).

To further investigate the effect of acceptor Q_π_ strength on BHJ blend energetics, we investigate the HOMO changes in PM6 and PBDB-T upon blending with various NFAs (IDIC, ITIC-series, Y6) with increasing Q_π_. As shown in Fig. 5, all NFAs have positive Q_π_ shifting the donor HOMO downwards upon blending. A positive correlation is observed between the magnitude of polymer HOMO deepening and the single-molecule Q_π_ of the NFA (DFT simulated). Similar trends are also observed in P3HT:NFA BHJs (see Supplementary Fig. 22). This helps to understand some systematic effects achieved by NFA structure engineering. For example, end group modification for ITIC derivatives results in an increase of Q_π_ from electron-donating (e.g., methyl in ITIC-2M) to electron-withdrawing groups (e.g., fluorine in ITIC-2F) and an increase in PM6 HOMO deepening (from 53 meV for ITIC-2M to 93 meV for ITIC-2F)^22^. This can aid in maintaining high *V*_OC_ in OPVs despite the LUMO deepening of the halogenated NFA. We note the correlation between the magnitude of HOMO deepening and NFA Q_π_ is not linear, which is most likely due to the variation in blend film morphology similar to the case of PM6:Y6 CF and CB blend films discussed in the previous section. However, the general trend is following the NFA Q_π_ which is one of the dominating factors in determining the energetic shift.

## Discussion

In this study, we have investigated neat NFA films as well as bilayer and BHJ films to elucidate a unified understanding on the correlation between molecular quadrupole moments and orientation, energetics and OPV performance. In neat NFA films, the bulk material energy levels depend strongly on the sign of the molecular quadrupole moments and its orientation with respect to the substrate. In bilayer films, the difference in Q_π_ between donor and acceptor and their relative molecular orientations determine the magnitude of interfacial band bending. Although it is most clearly seen in PHJs, an impact is still observed in BHJs. The generality of our conclusion is confirmed by measuring quadrupole-induced energetic shift in other polymer:NFA systems.

The morphology-dependent energetic shifts reported herein have a significant impact on OPV devices, as they directly change the electronic bandgaps in both bilayer and BHJ devices. At the same time, the extent of quadrupole-induced band bending at the D/A heterointerface alters the charge carrier distribution and the rate of non-geminate recombination. Considering both energetic and kinetic losses associated with the morphology change allows us to achieve a more consistent and in-depth understanding of *V*_OC_ losses in NFA-based OPV devices. The extent of interfacial band bending as well as the presence of domains with mixed orientations was also found to affect exciton and CT state dissociation critical for charge generation efficiency.

In summary, this study highlights the importance of considering orientation-dependent energetic shifts in high performance OPVs resulting from the large molecular quadrupole moments of NFAs. By controlling molecular quadrupole moments via changes in the chemical structure and fine-tuning of the thin film morphology, both energetics and recombination kinetics could be optimized to further improve the *V*_OC_ of OPVs while maintaining high efficiency of charge generation.

## Methods

### Materials and sample preparation

ITO substrates were sequentially cleaned by ultrasonication with detergent, distilled water, acetone and isopropanol. Zinc oxide (ZnO) precursor solution was prepared by sol-gel method, dissolving zinc acetate dihydrate (0.11 g mL^−1^) in 2-methoxyethanol with 3 vol% ethanolamine and sonicating for over 2 h. The solution was spun on ITO and subsequently annealed in air at 200 °C for 20 min. Y6 (Organtec) was dissolved in chloroform (CF, 8 mg mL^−1^) and chlorobenzene (CB, 10 mg mL^−1^). Y6 solutions were spin-cast on top of the substrates and annealed at 100 °C for 5 min in a N_2_-filled glove box. PM6 (Organtec) was dissolved in CB:1,8-diiodooctane (99:1 volume ratio) with a concentration of 20 mg mL^−1^. Droplets (15 μL) of PM6 solutions formed pristine films on distilled water, and then films were transferred on top of the Y6 layer by stamping the Y6-ZnO substrate gently on the floated PM6 film^29,30^. Samples were annealed at 100 °C for 10 min in the N_2_-filled glove box. To fabricate BHJ films, PM6:Y6 blend solution (1:1.2 weight ratio, 14 mg mL^−1^ in CF and 0.5 vol% 1-chloronaphthalene, or 20 mg mL^−1^ in CB) was spin-coated on the ZnO layer, then annealed at 100 °C for 10 min. For devices, top contacts were deposited by thermal evaporation in vacuum (10^−6^–10^−7^ mbar), using 10 nm thick MoO_3_ for the hole extraction layer and 100 nm Ag as anode. The area of the electrode defined an active area of 6 mm^2^. A similar procedure was followed to deposit other combinations of NFAs (ITIC, Sigma Aldrich; IDIC and Y6, Organtec; ITIC-2M and ITIC-2F, synthesized by collaborators) with different polymers (PM6 and PBDB-T, Organtec; P3HT,> 99% regioregular, average molecular weight of 60 k, Sigma Aldrich) in 1:1 blend ratio and 20 mg mL^−1^ dilution.

### Photovoltaic characterization

*J-V* characteristics of solar cells were collected with a Keithley 2400 source (calibrated with a certified reference silicon cell) measurement under AM 1.5 G illumination at 100 mW cm^−2^ using a xenon lamp (Oriel Instruments) with nitrogen encapsulated chamber. External quantum efficiency (EQE) measurements were performed using a Bentham PVE300 with monochromatic light from a dual xenon/quartz halogen light source with nitrogen encapsulated chamber.

### Transient photovoltage (TPV) and transient photocurrent (TPC)

A ring of 12 white light LEDs was used to provide the background light intensity for the TPV measurements. The 1-sun equivalent light intensity was calibrated by matching the *J*_SC_ and *V*_OC_ to the values obtained for AM1.5 G and a photodiode is used to calibrate the LED output to the light intensity range used (here ~ 1% sun to 600% sun). The small perturbation light pulse was provided by a Continuum Minilite Nd:YAG laser with a 532 nm wavelength and a 5 ns pulse. For the TPV measurement, the anode is connected to a Tektronix TDS3032B oscilloscope using the 1 MΩ input impedance to measure the voltage transient while the cathode is connected to the ground. The small perturbation lifetime (${{{{{{\rm{\tau }}}}}}}_{{{{{{\rm{TPV}}}}}}}$) is obtained by fitting a monoexponential decay to the TPV transient and the total carrier lifetime is calculated using the formula described^49^. To correct the TPV lifetime for capacitive effects, the active layer lifetime was calculated by using the formula ${{{{{{\rm{\tau }}}}}}}_{{{{{{\rm{AL}}}}}}}={{{{{{\rm{\tau }}}}}}}_{{{{{{\rm{TPV}}}}}}}\left(\frac{{{{{{{\rm{C}}}}}}}_{{{{{{\rm{AL}}}}}}}}{{{{{{{\rm{C}}}}}}}_{{{{{{\rm{AL}}}}}}}+{{{{{{\rm{C}}}}}}}_{{{{{{\rm{geo}}}}}}}}\right)$^60^. For the TPC measurements, the device is kept at low background light intensities (dark to 25% sun) and the current transient is measured by connecting the cathode to the 1 MΩ input impedance via a 50 Ω resistor and grounding the anode. The voltage transient measured across the resistor is converted into a current using Ohm’s law and integration yields the total amount of charge carriers generated by the laser pulse. The differential capacitance is calculated as reported previously^56^. The differential capacitance is corrected for the geometric capacitance before using the numerical integration as detailed in the above reference to yield the corrected active layer charge carrier density.

### Energetic characterization and surface photovoltage (SPV)

APS04 (KP Technology) system was used to carry out the ambient photoemission spectroscopy (APS), surface photovoltage (SPV) and dark work function (DWF) measurements. DWF measurement was carried out first, followed by SPV and APS to minimize potential degradations of samples. DWF measurement utilized the off-null Kelvin probe technique where a 2 mm gold tip was applied with an oscillating bias of 7 V. The DWF of the tip was calibrated from the contact potential difference (CPD) and valence band edge of a silver reference. The DWF of each sample was determined by the CPD between the sample and the tip. For SPV measurements, the samples were periodically illuminated by a quartz tungsten halogen lamp with an emission spectrum between 400 to 800 nm and power of 20 mW cm^−2^ while the change in CPD was monitored by Kelvin probe. For APS measurements, the samples were illuminated by UV light with its photon energy ranged from 4.5 to 6.8 eV to generate photoelectrons. The photoelectrons and the generated radicals were then collected by a positively biased tip^27^. The HOMO level was then obtained via linear fitting the cube root of the photoemission intensity. The density of states was derived from the derivative of the photoemission to energy following Fowler’s theory^61^.

### Morphology characterization

ParkNX10 atomic force microscope (AFM) system with Park silicon PPP-NCHR tips was used for topography characterization of neat, bilayer and BHJ samples. Measurements were carried out using non-contact mode with piezoelectric tip oscillating at a fixed frequency and amplitude at a set distance of around 10 nm above the sample surface. Tip scans over 256 pixels across a 5 μm range at a rate of 0.5 Hz. A 5 × 5 μm image was obtained for each sample and the root-mean-square (RMS) roughness was obtained using the Gwyddion software package. GIWAXS measurements were conducted at the PLS-II 9 A U-SAXS beamline of the Pohang Accelerator Laboratory.

### Simulations

Density functional theory (DFT) at the B3LYP level was done by using Gaussian16 software with a basis set of 6-311 G(d,p)^62^. The molecules were optimized to their ground state in order to obtain the ground state geometry, quadrupole moment and ESP map. For polymers, their corresponding trimers were used as a characteristic representation. The ESP was mapped to the electron density for visualization. The HOMO/LUMO energies of the Y6 molecules in face-on and edge-on packing structures were calculated by DFT and MD calculations. The packing structures were optimized by DFT calculation using Vienna ab initio simulation package (VASP)^63–68^. Here the DFT calculations were performed for 8 molecules in a cell with periodic boundary conditions under the condition of flexible cell. After roughly building the face-on and edge-on packing structures by referring to papers reported by Lei Zhu et al.^31^ and Weigang Zhu et al.^59^, the initial structures of DFT calculations were generated by MD relaxations using Desmond package (Schrödinger, LLC, NY, USA)^69^ with a force field of Optimized Potentials for Liquid Simulations (OPLS) which has been widely used for organic systems. The initial and final MD geometry are in Supplementary Data 1 and 2). To study the effect of quadrupole moment, the DFT calculations for each Y6 molecule extracted from the face-on and edge-on packings were carried out by Jaguar DFT package (Schrödinger, LLC, NY, USA)^70^ with a basis set of 6-31 G**/B3LYP, taking into account electrostatic potentials induced by the ESP charges on surrounding Y6 molecules. The geometry used for face-on and edge-on are in Supplementary Data 3 and 4). To study the effect of intermolecular electronic coupling, DFT calculations were performed on 10 dimer structures randomly extracted from the MD-simulated face-on and edge-on packing structures.

### Raman spectroscopy

Raman spectra were acquired by a Renishaw inVia Raman microscope in backscattering configuration using a 514 nm laser excitation (Argon, Titanium Sapphire laser, 10 µm spot diameter, 25% defocusing on samples). The samples were constantly exposed to a nitrogen flow in a Linkam chamber to avoid degradation. Diffracted light was separated by a diffraction grid (2400 lines/mm). The spectrometer was calibrated by a Si reference sample. Acquisition parameters such as laser power, exposure time and measurement accumulation number were optimized, and spectra were measured over multiple positions on the sample to improve accuracy. Polynomial fitting was used for PL background removal.

### Transient absorption spectroscopy

Helios spectrometer (Spectra-Physics, Newport Corp.) was used to measure the broadband pump-probe femtosecond-TA spectra and kinetics for thin film samples. Ultrafast laser pulses (800 nm, 100 fs duration) were generated by a 1 kHz Ti:sapphire regenerative amplifier (Solstice, Spectra-Physics, Newport Corp.). One portion of the 800 nm pulse was directed to an optical parametric amplifier (TOPAS Prime, Spectra-Physics) and a frequency mixer (Niruvis, Light Conversion) to tune the visible pump pulses at various wavelengths. The pump pulses were modulated at a frequency of 500 Hz by a mechanical chopper. The rest of the 800 nm pulse was routed onto a mechanical delay stage with a 6 ns time window and directed through a YAG for the near-infrared region. The probe pulse was split into two by a neutral density filter. One portion of the probe pulse served as the reference and was directly sent to the fiber-optic coupled multichannel spectrometers (CCD and InGaAs sensors). The rest of the probe pulse together with the pump pulse were focused onto the same spot on the samples with a beam size of around 0.5 mm^2^ before sending it to the spectrometer.

### Reporting summary

Further information on research design is available in the Nature Portfolio Reporting Summary linked to this article.

## Supplementary information

Supplementary Information

Description of Additional Supplementary Files

Supplementary Data 1

Supplementary Data 2

Supplementary Data 3

Supplementary Data 4

Solar Cells Reporting Summary

## Data Availability

The data supporting the findings of this study are available from the corresponding authors upon request.
