# Supplementary material for: Molecular orientation-dependent energetic shifts in solution-processed non-fullerene acceptors and their impact on organic photovoltaic performance
Source: Nat Commun. 2023 Apr 4;14:1870. doi: 10.1038/s41467-023-37234-0 (PMC10073232; doi:10.1038/s41467-023-37234-0)
Supplement: Supplementary file 1 — Supplementary Information [file 41467_2023_37234_MOESM1_ESM.pdf]

Supplementary Information

Molecular orientation-dependent energetic shifts in solution-processed non-fullerene acceptors and their impact on organic photovoltaic performance

Yuang Fu[1,6†], Tack Ho Lee[2,7†], Yi-Chun Chin[1†], Richard A. Pacalaj[2], Chiara Labanti[1], Song Yi Park[1], Yifan Dong[2], Hye Won Cho[3], Jin Young Kim[3,8], Daiki Minami[4*], James R. Durrant[2,5*], & Ji-Seon Kim[1*]

[1]Department of Physics and Centre for Processable Electronics, Imperial College London, London SW7 2AZ, UK

E-mail: ji-seon.kim@imperial.ac.uk

[2]Department of Chemistry and Centre for Processable Electronics, Imperial College London, London W12 0BZ, UK

E-mail: j.durrant@imperial.ac.uk

[3]School of Energy and Chemical Engineering, Ulsan National Institute of Science and Technology (UNIST), Ulsan 44919, Republic of Korea

[4]CSE team, Innovation Center, Samsung Electronics, Co. Ltd., 1 Samsungjeonja-ro, Hwaseong-si, Gyeonggi-do 18448, Republic of Korea

E-mail: daiki.minami@samsung.com

[5]SPECIFIC IKC, Department of Materials, University of Swansea, Bay Campus, Swansea SA1 8EN, UK.

[6]Present address: Department of Physics, The Chinese University of Hong Kong, New Territories, Hong Kong 999077, China

[7]Present address: Department of Chemistry Education, Graduate Department of Chemical Materials, Institute for Plastic Information and Energy Materials, Sustainable Utilization of Photovoltaic Energy Research Center/Engineering Research Center, Pusan National University, Busan 46241, Republic of Korea

[8]Graduate School of Carbon Neutrality, Ulsan National Institute of Science and Technology (UNIST), Ulsan 44919, Republic of Korea

*Corresponding authors

[†]These authors contributed equally

Supplementary Figures

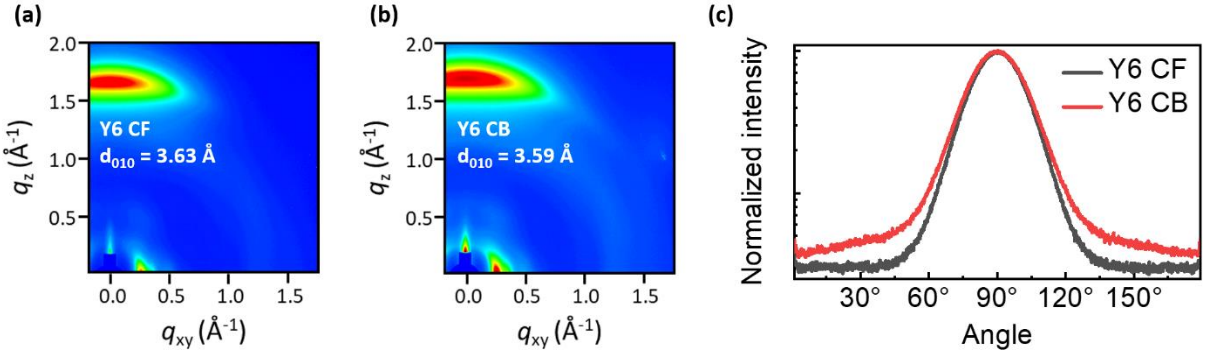

Supplementary Fig. 1. GIWAXS measurements of Y6 neat films. 2D GIWAXS patterns of **(a)** Y6 CF and **(b)** Y6 CB with π-π stacking distance (d_{010}) along the out-of-plane direction highlighted. The crystal coherence lengths along the π-π stacking direction are calculated to be 33.0 and 31.9 Å for Y6 CF and CB, respectively. **(c)** Normalized polar angle distribution of π-π stacking peak. The ratio of edge-on to face-on distribution was calculated by the area of angular linecuts (measured intensity) at 0°~45° and 45°~90° for edge-on and face-on, respectively. The ratio of edge-on to face-on area is 0.09

and 0.12 for Y6 CF and Y6 CB, respectively, indicating that Y6 CB has wider polar angle distribution and more edge-on oriented molecules.

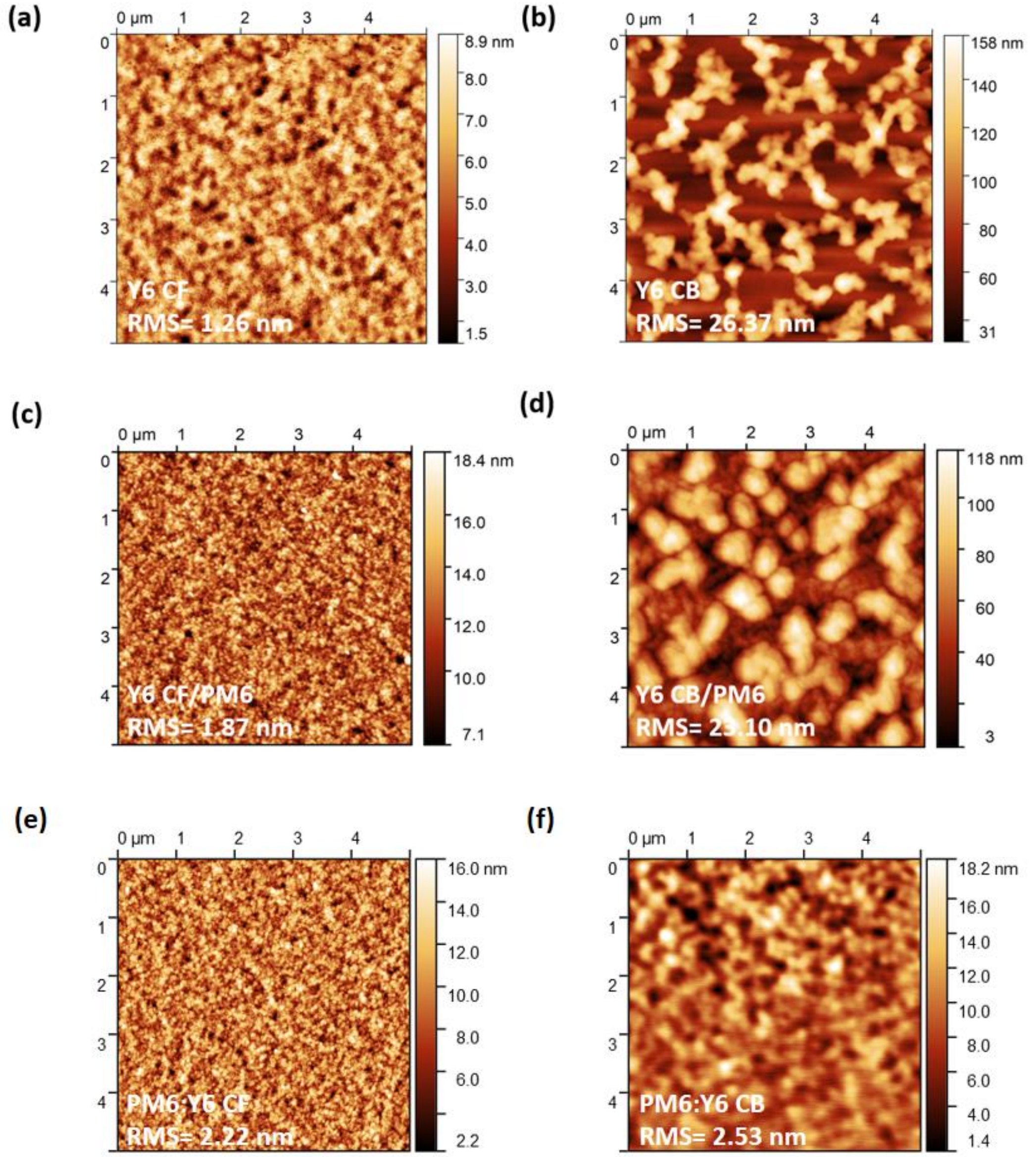

Supplementary Fig. 2. Surface characterizations of neat, bilayer and blend films. Topography of **(a)** Y6 CF and **(b)** Y6 CB neat films, **(c)** Y6 CF/PM6 and **(d)** Y6 CB/PM6 bilayers and **(e)** PM6:Y6 CF and **(f)** PM6:Y6 CB blend films, measured using atomic force spectroscopy with root-mean-square (RMS) roughness labelled as inset.

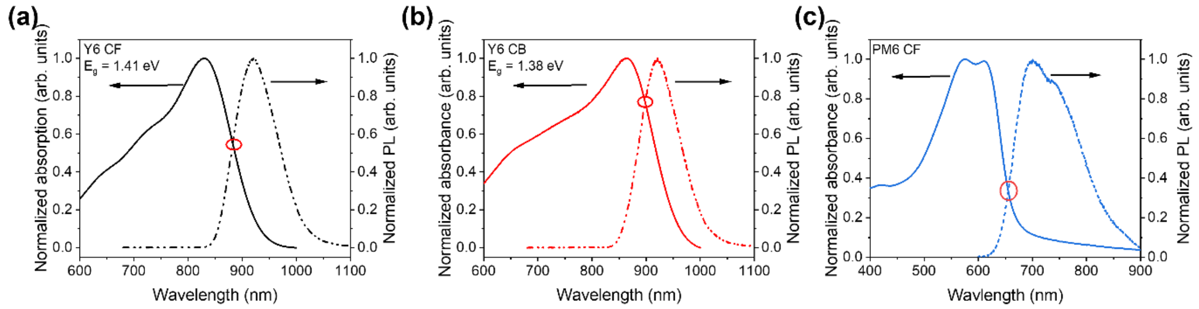

Supplementary Fig. 3. Optical characterizations of neat films. Normalized absorbance and PL spectra for **(a)** Y6 CF, **(b)** Y6 CB and **(c)** PM6 CF. The optical bandgap (E_g) is determined from the intersection point, as highlighted in red.

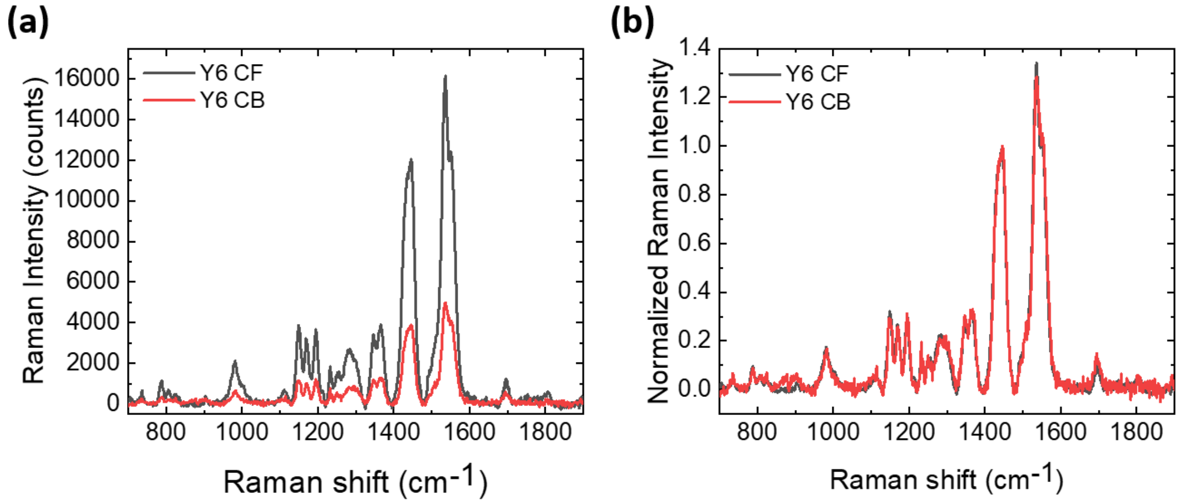

Supplementary Fig. 4. Resonant Raman measurements of Y6 neat films. (a) Raw and **(b)** normalized resonant Raman spectra of Y6 CF and Y6 CB deposited on ITO/ZnO, excited at 633 nm.

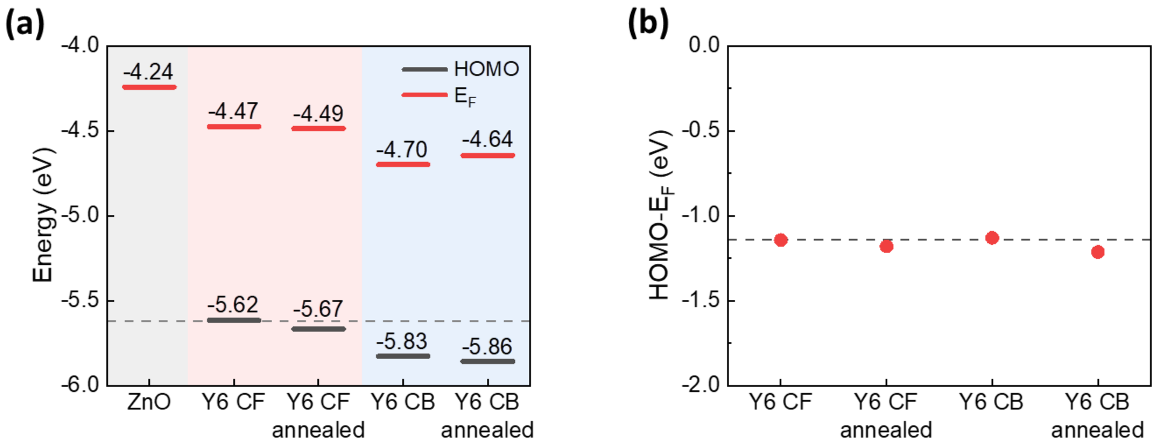

Supplementary Fig. 5. Energetics of Y6 neat film. (a) HOMO and Fermi level (E_F) depending on Y6 preparation conditions. Films are annealed at 100 °C in 10 mins. **(b)** Similar energy gap between HOMO and Fermi level depending on Y6 preparation conditions, showing the nature of the electrostatic effect from quadrupole moment.

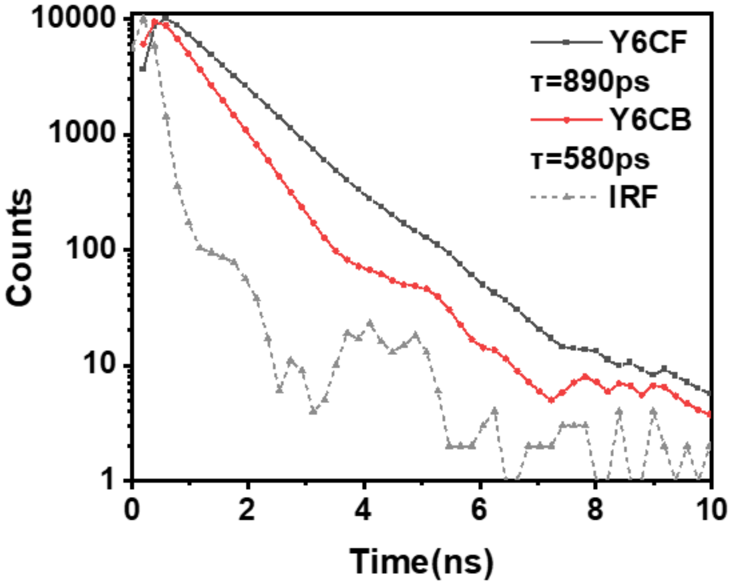

Supplementary Fig. 6. Exciton dynamics of Y6 neat films. TCSPC of Y6 CF and Y6 CB neat films deposited directly on top of quartz without ZnO interlayer. The decay lifetime is fitted using a single exponential function. The grey lines indicate the prompt decay (instrument response function-IRF), which induces artefacts in the decay curve of Y6 CB.

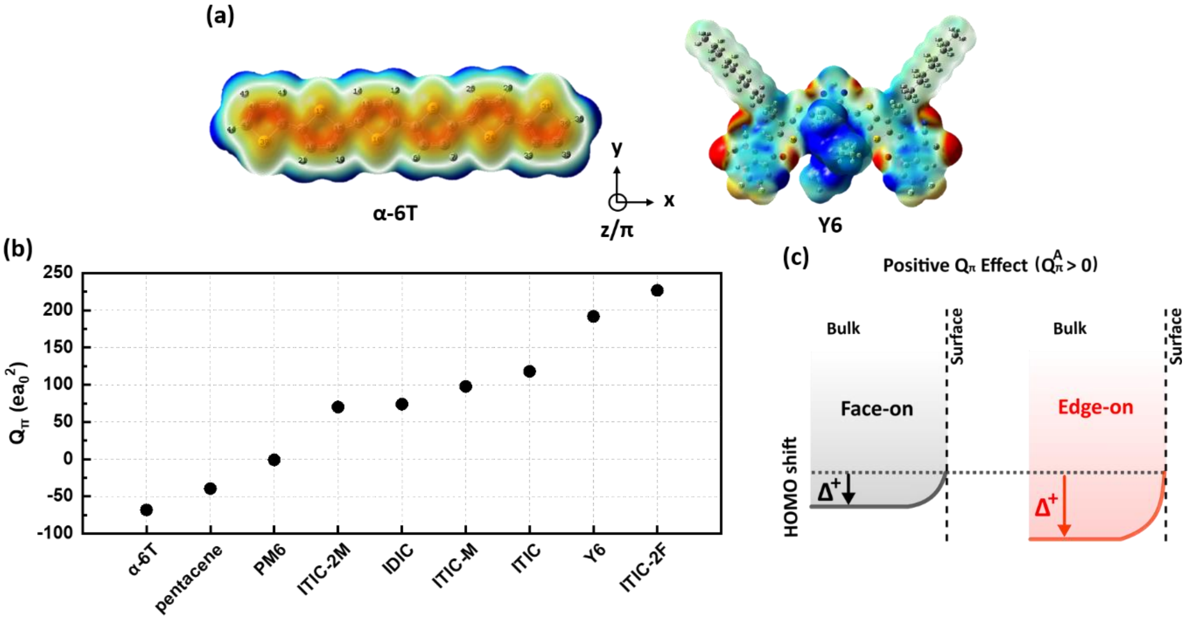

Supplementary Fig. 7. Simulated molecular quadrupole moments and schematics of interfacial band bending. (a) Electrostatic potential (ESP) of α-6T and Y6 molecules because of uneven charge distribution along conjugated backbones. **(b)** Molecular quadrupole moments of NFAs and small molecular donors (and polymer PM6) along π–π stacking direction (Q_π) obtained via gas-state DFT. **(c)** Schematics showing the impact of the positive quadrupole moment on the acceptor energetics in bulk and interfacial regions depending on the molecular orientation. At the boundary of the crystals (i.e. film/air interface), this electrostatic energetic shift will diminish, causing an upward band bending (Δ^+) of the Y6 energy levels originating from its positive Q_π value.

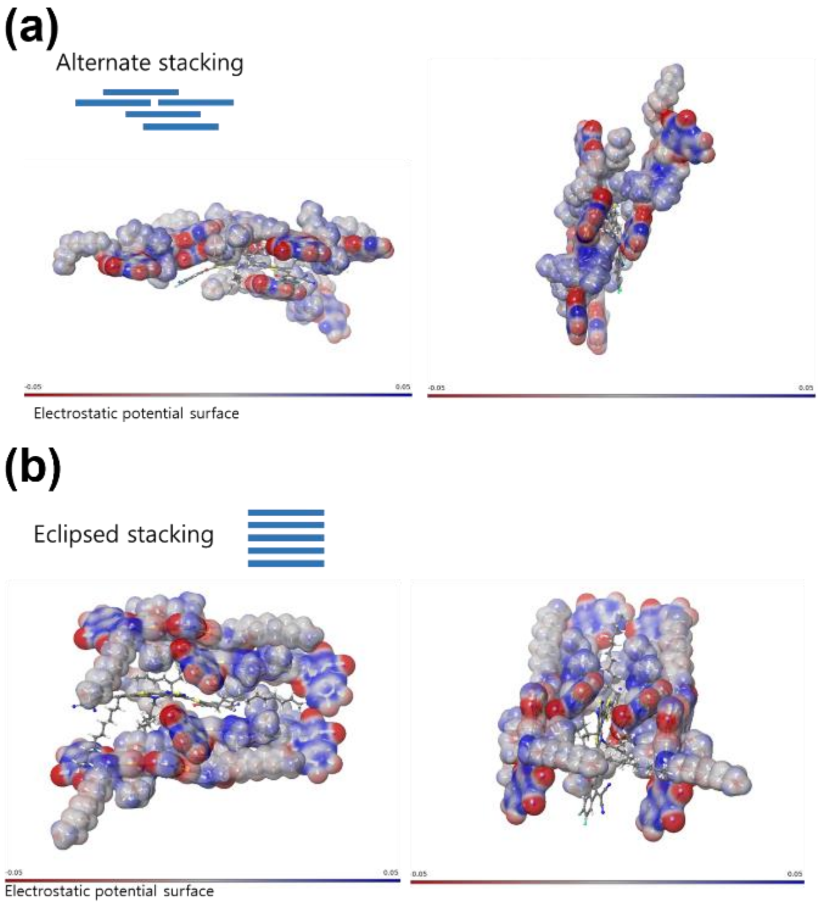

Supplementary Fig. 8. Molecular dynamics (MD) simulations. MD-simulated structures in **(a)** face-on and **(b)** edge-on packed Y6 molecules. The simplified schematics on the top left highlight their different stacking patterns. The electrostatic potential surfaces of surrounding molecules are visualized to illustrate the effect of partial charges on the energy levels of the centre molecules.

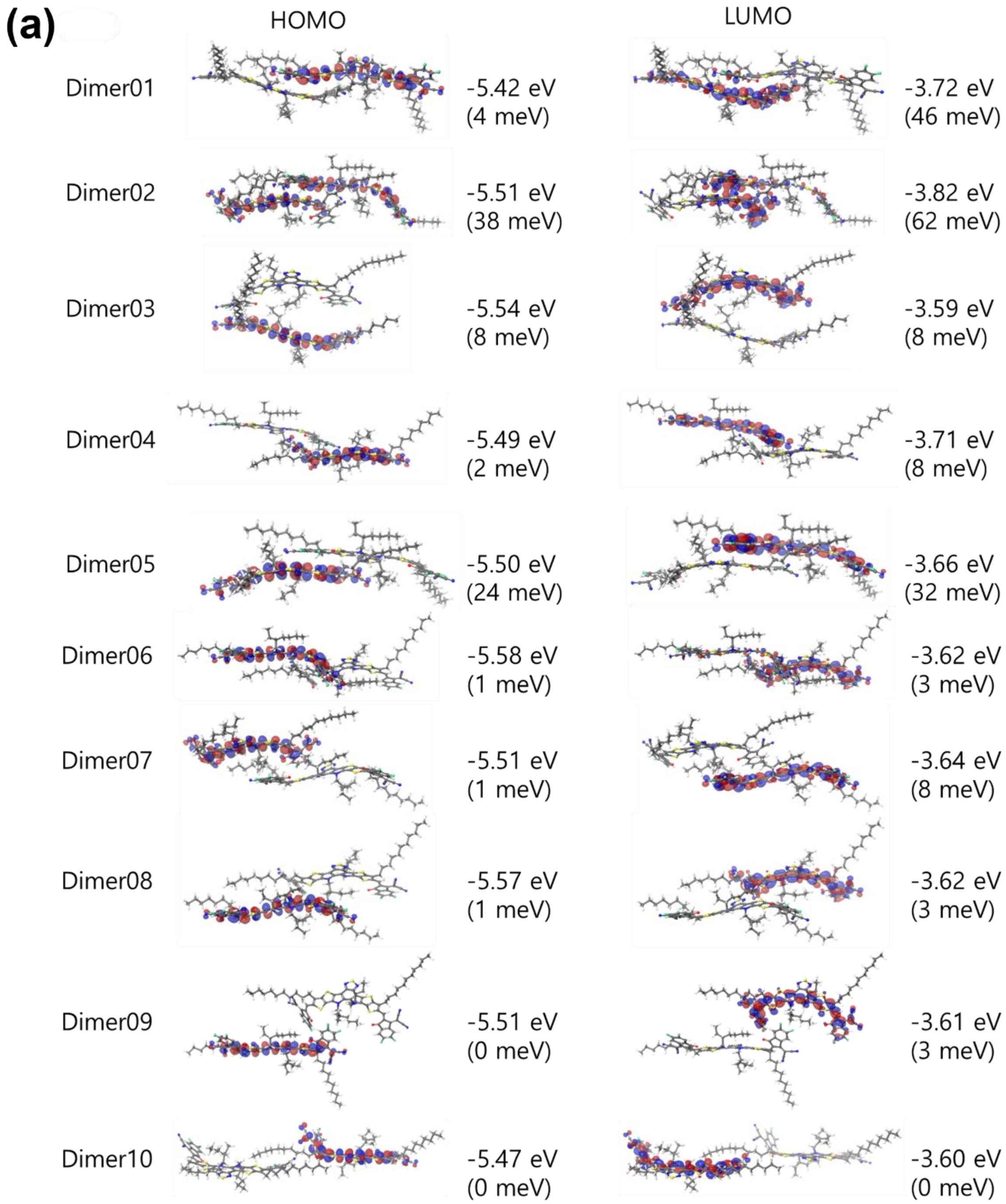
(a)
HOMO
LUMO
Dimer01
-5.42 eV
(4 meV)
-3.72 eV
(46 meV)
Dimer02
-5.51 eV
(38 meV)
-3.82 eV
(62 meV)
Dimer03
-5.54 eV
(8 meV)
-3.59 eV
(8 meV)
Dimer04
-5.49 eV
(2 meV)
-3.71 eV
(8 meV)
Dimer05
-5.50 eV
(24 meV)
-3.66 eV
(32 meV)
Dimer06
-5.58 eV
(1 meV)
-3.62 eV
(3 meV)
Dimer07
-5.51 eV
(1 meV)
-3.64 eV
(8 meV)
Dimer08
-5.57 eV
(1 meV)
-3.62 eV
(3 meV)
Dimer09
-5.51 eV
(0 meV)
-3.61 eV
(3 meV)
Dimer10
-5.47 eV
(0 meV)
-3.60 eV
(0 meV)

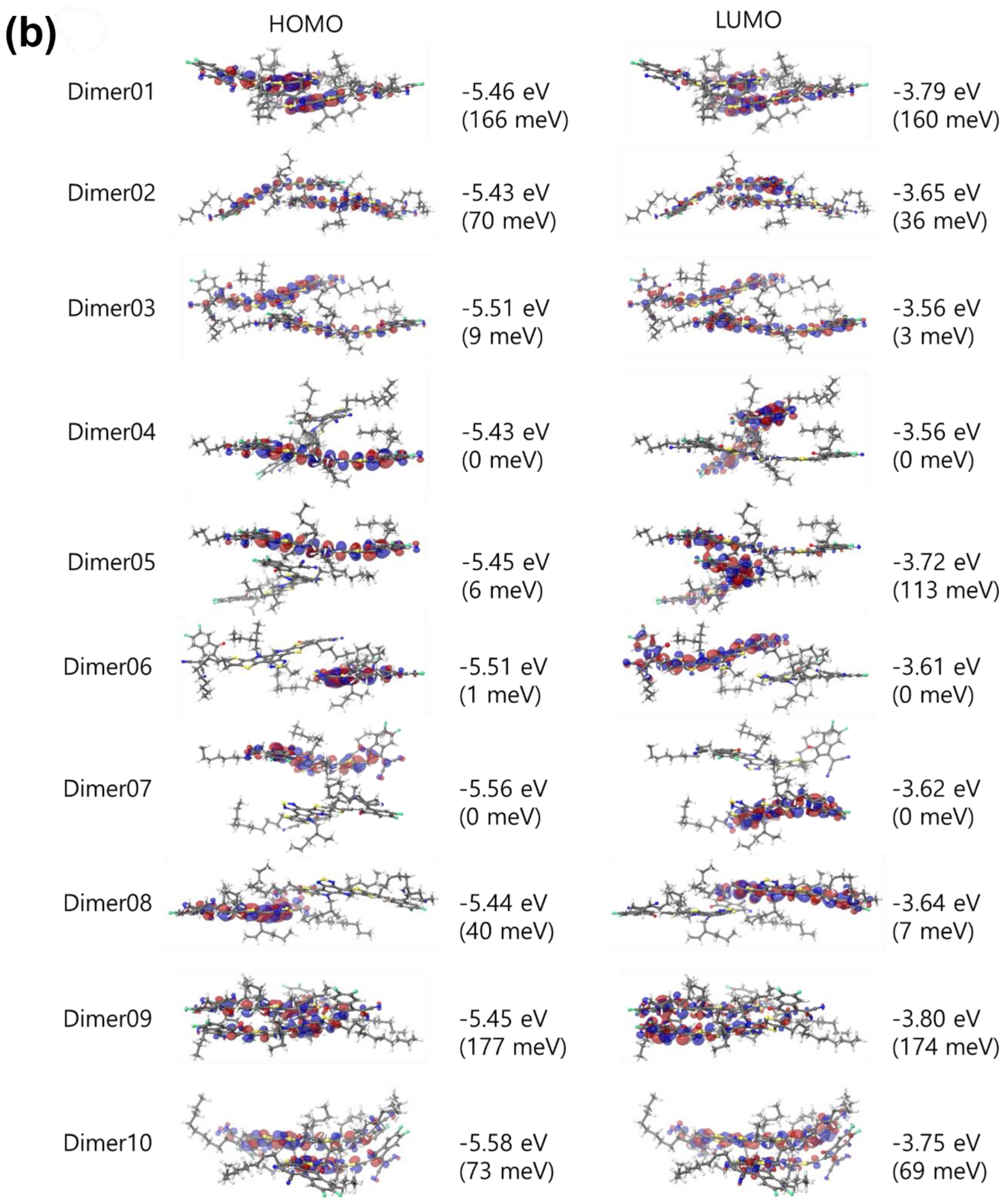
(b)
HOMO
LUMO
Dimer01
-5.46 eV
(166 meV)
-3.79 eV
(160 meV)
Dimer02
-5.43 eV
(70 meV)
-3.65 eV
(36 meV)
Dimer03
-5.51 eV
(9 meV)
-3.56 eV
(3 meV)
Dimer04
-5.43 eV
(0 meV)
-3.56 eV
(0 meV)
Dimer05
-5.45 eV
(6 meV)
-3.72 eV
(113 meV)
Dimer06
-5.51 eV
(1 meV)
-3.61 eV
(0 meV)
Dimer07
-5.56 eV
(0 meV)
-3.62 eV
(0 meV)
Dimer08
-5.44 eV
(40 meV)
-3.64 eV
(7 meV)
Dimer09
-5.45 eV
(177 meV)
-3.80 eV
(174 meV)
Dimer10
-5.58 eV
(73 meV)
-3.75 eV
(69 meV)

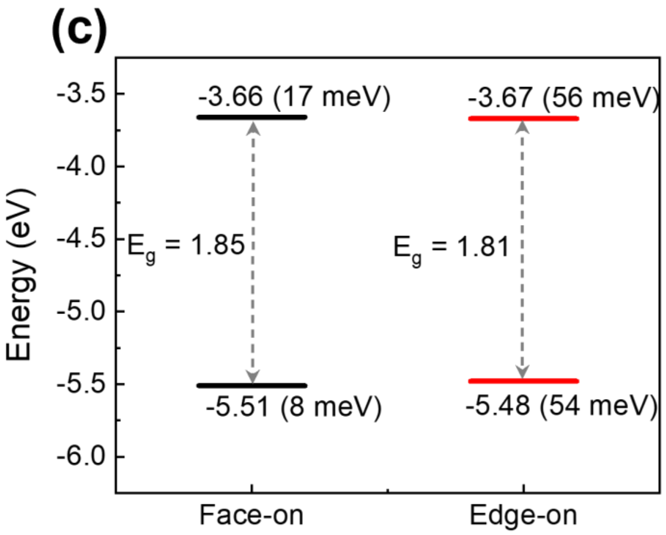

Supplementary Fig. 9. DFT-simulated electronic couplings and energy levels of Y6 dimer pairs. 10 dimer structures randomly extracted from the MD-simulated stacking structures in (a) face-on and (b) edge-on packed Y6 molecules with their respective HOMO/LUMO energy levels and electronic coupling values calculated using DFT without considering electrostatic interactions. The average energy levels and electronic coupling values (in brackets) are shown in (c). Similar bandgaps (E_g) are highlighted.

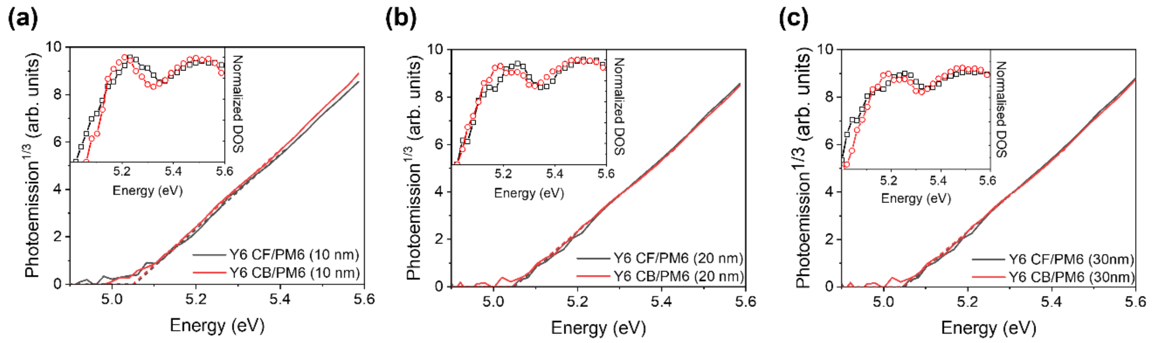

Supplementary Fig. 10. Energetics of bilayer film. APS spectra of **(a)** 10 nm, **(b)** 20 nm, and **(c)** 30 nm PM6 layer deposited on top of Y6 CF and Y6 CB. The inset is the normalized density of states.

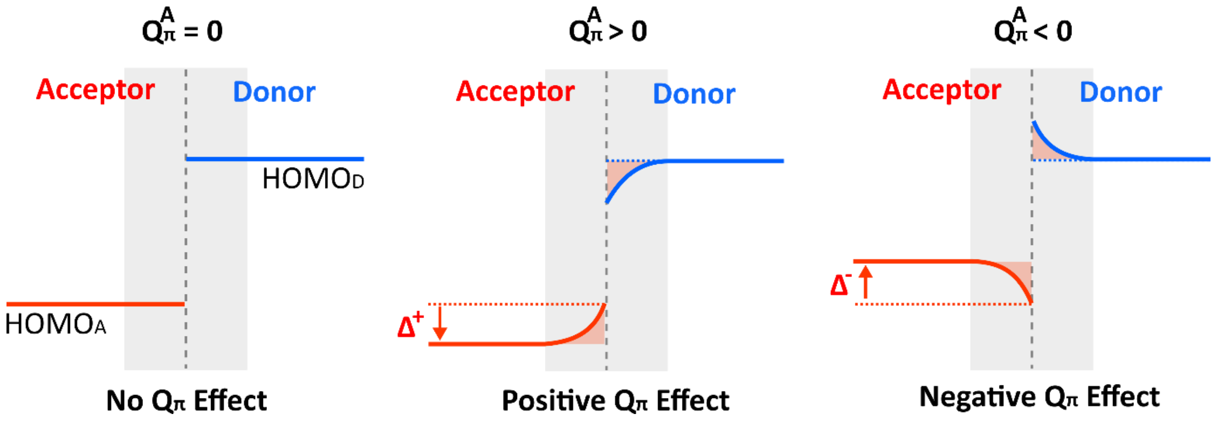

Supplementary Fig. 11. Impact of acceptor quadrupole moment (Q_π^A) on the donor-acceptor interface. From left to right: no quadrupole moment, positive quadrupole moment and negative quadrupole moment. The positive/negative quadrupole moment deepens/shallows the HOMO level of acceptor and the influence tails off on the interface. The quadrupole moment effect also shifts the HOMO level of donor on the interface.

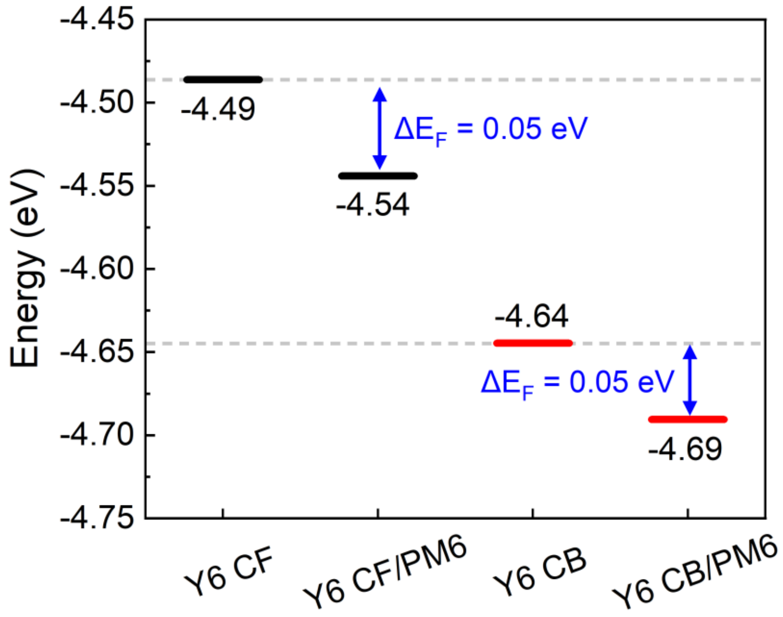

Supplementary Fig. 12. Vacuum level shift at the D:A heterointerface of bilayer films. Fermi level shifts (ΔE_F) from Y6 CF to Y6 CF/PM6 (5 nm) and from Y6 CB to Y6 CB/PM6 (5 nm) demonstrate only small vacuum level shifts at the Y6/PM6 interface.

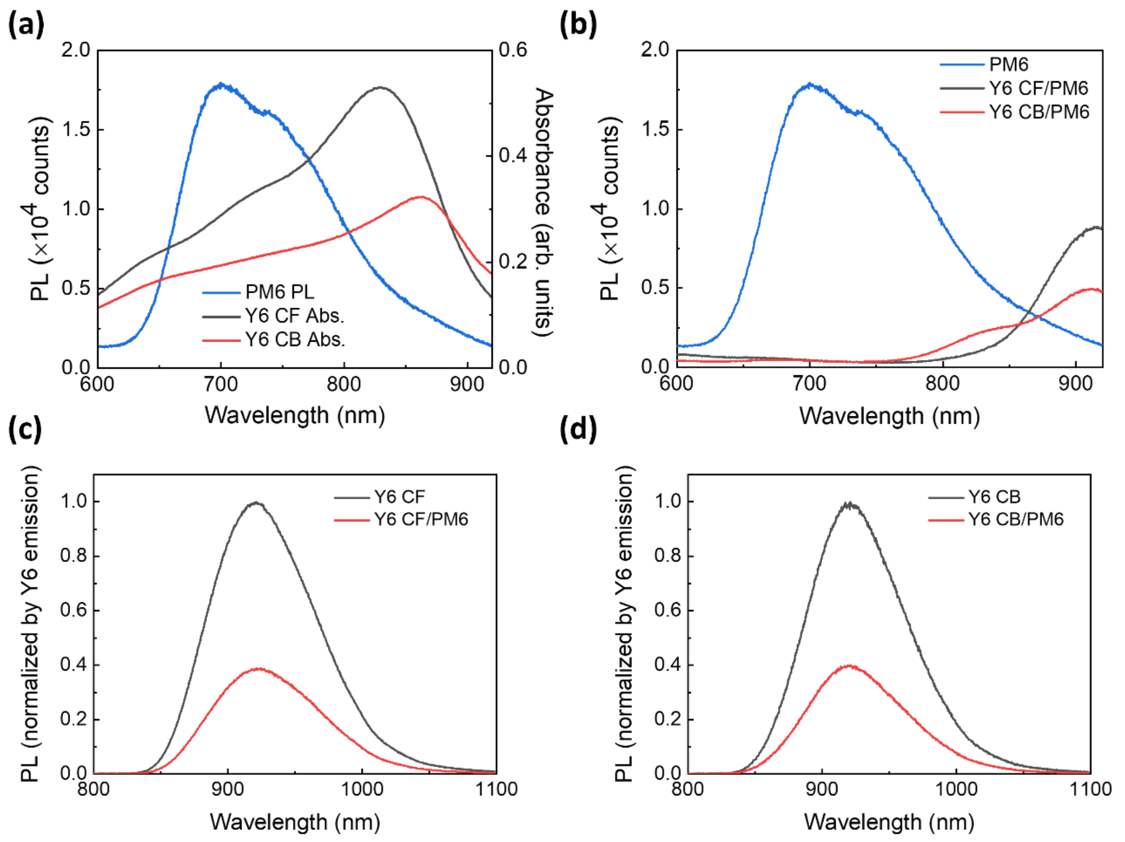

Supplementary Fig. 13. PL quenching measurements of bilayer films. (a) Photoluminescence (PL) of neat PM6 film (excited at 405 nm) and absorption spectra of neat Y6 CF and neat Y6 CB. **(b)** PL spectra of neat PM6, Y6 CF/PM6, and Y6 CB/PM6 excited at the wavelength of 405 nm. PL spectra of **(c)** neat Y6 CF and Y6 CF/PM6, and **(d)** neat Y6 CB and Y6 CB/PM6 excited at the wavelength of 780 nm. For **(c)** and **(d)**, PL intensity was normalized with respect to that of the respective Y6 neat film.

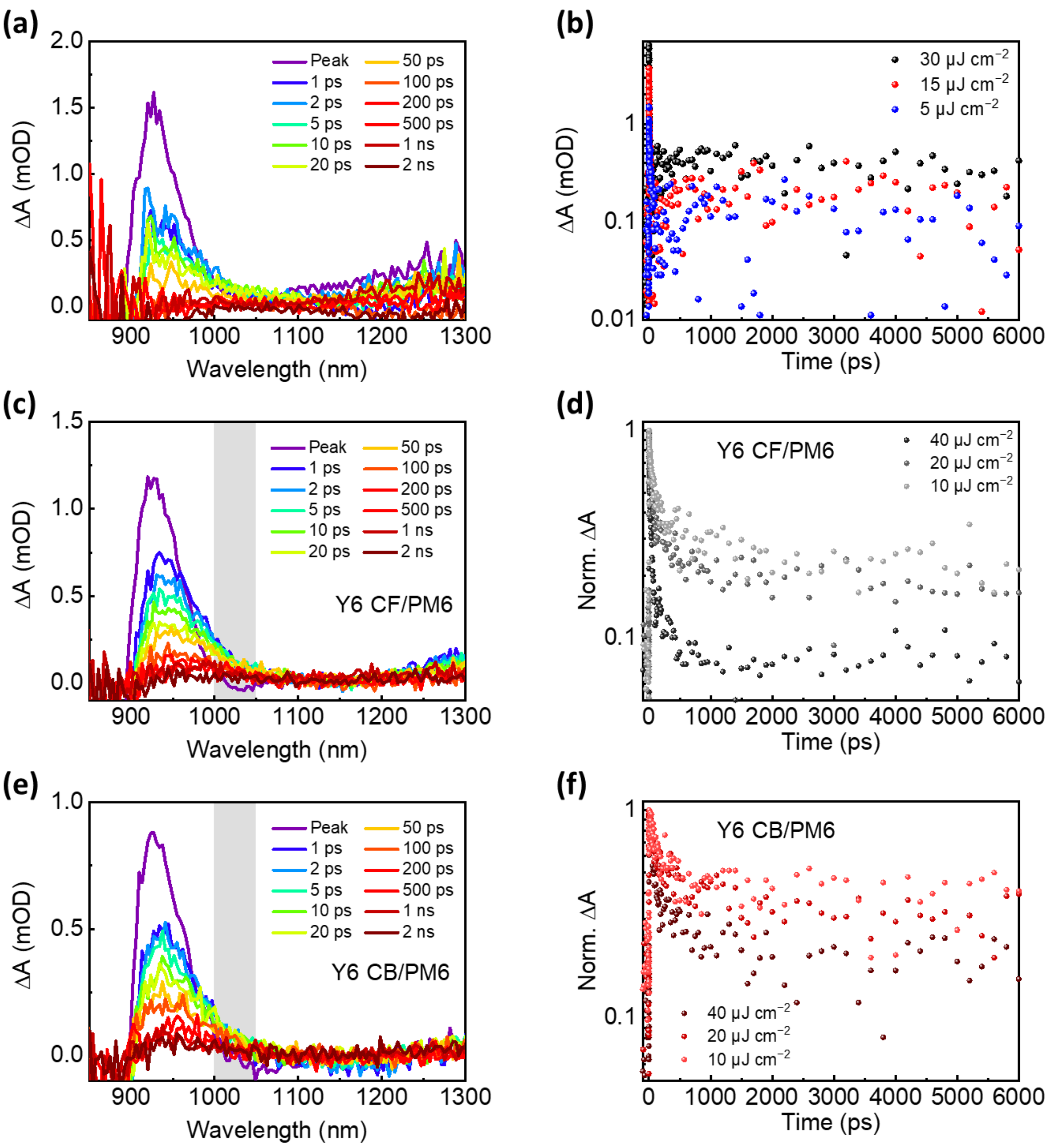

Supplementary Fig. 14. Transient absorption (TA) measurements. TA spectra of **(a)** Y6 CF, **(c)** Y6 CF/PM6 and **(e)** Y6 CB/PM6 film in near-infrared probe region excited at 775 nm. Corresponding TA decay dynamics of **(b)** Y6 at the probe wavelength of 920-930 nm, **(d)** Y6 CF/PM6 and **(f)** Y6 CB/PM6 at the probe wavelength of 1000-1050 nm. Pump wavelength was 775 nm to selectively photoexcite Y6.

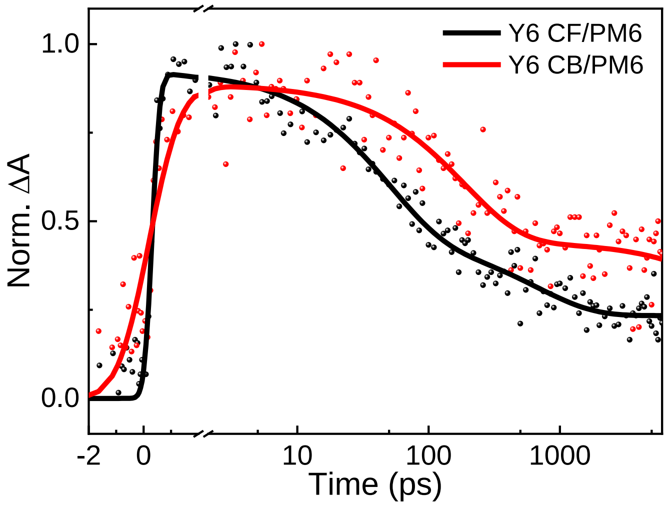

Supplementary Fig. 15. Charge carrier dynamics of bilayer films. Transient absorption decay dynamics for Y6 CF/PM6 and Y6 CB/PM6 films excited at 775 nm (fluence: 10 μJ cm^{-2}) and probed at 1000-1050 nm. Kinetics are fitted by one exponential rise and two exponential decays (solid lines).

***Carrier dynamic analyses from femtosecond TA measurements*:** Photoinduced absorption (PIA) peak of neat Y6 at 925 nm decays to zero within 100 ps in Supplementary Fig. 14 a and b, indicating that the exciton-exciton annihilation is finished by 100 ps in neat Y6 film. In both Y6/PM6 PHJs, PIA peaks are boarder with shoulders at 1000-1050 nm in Supplementary Fig. 14c and e. Corresponding pump fluence-dependent kinetics are presented in Supplementary Fig. 14d and f. The comparison of the recombination kinetics of Y6 CF/PM6 and Y6 CB/PM6 demonstrates suppressed recombination losses in Y6 CB/PM6 bilayers (Supplementary Fig. 15).

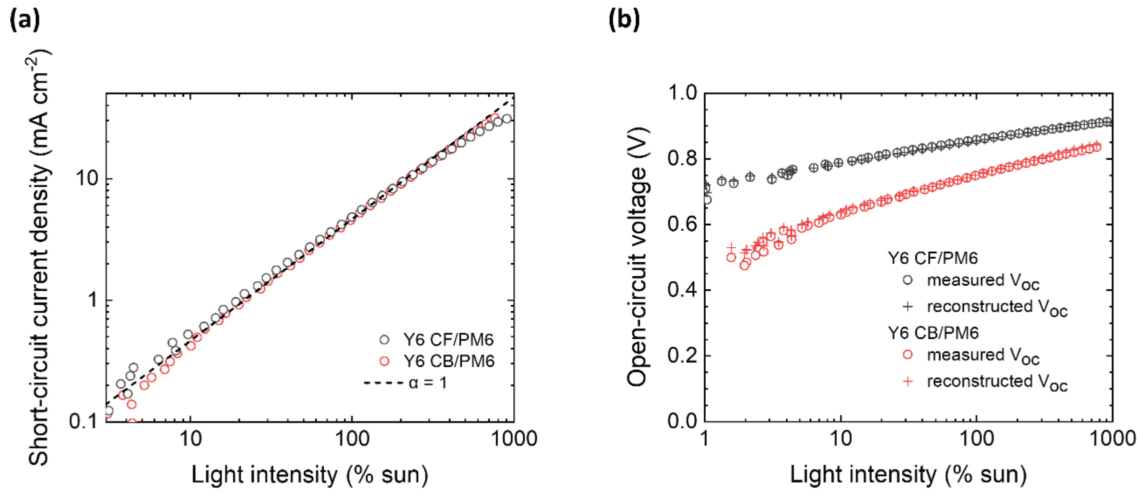

Supplementary Fig. 16. Light intensity-dependent measurements of bilayer devices. (a) short-circuit current density and **(b)** open-circuit voltage (V_{OC}) measured under a wide range of light intensities. The dashed line in (a) describing the case where $Jsc \propto$ light intensity ($\alpha = 1$)is to guide the eye. Reconstruction of the V_{OC} from our experimentally fitted charge carrier density and lifetime data are well matched to the measured V_{OC} in (b).

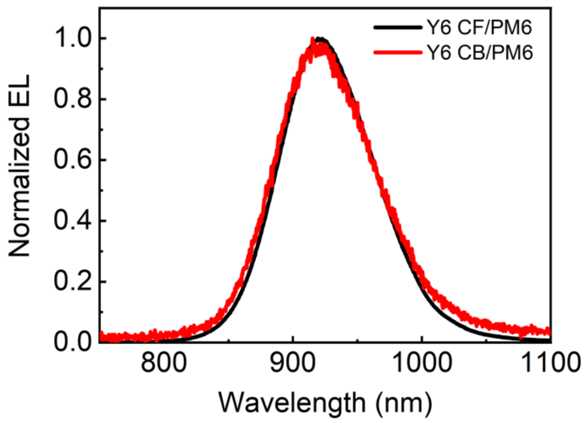

Supplementary Fig. 17. Electroluminescence spectra of bilayer devices. The measurements were taken under a forward bias of 1 V. The bottom Y6 layer was deposited from either CF or CB.

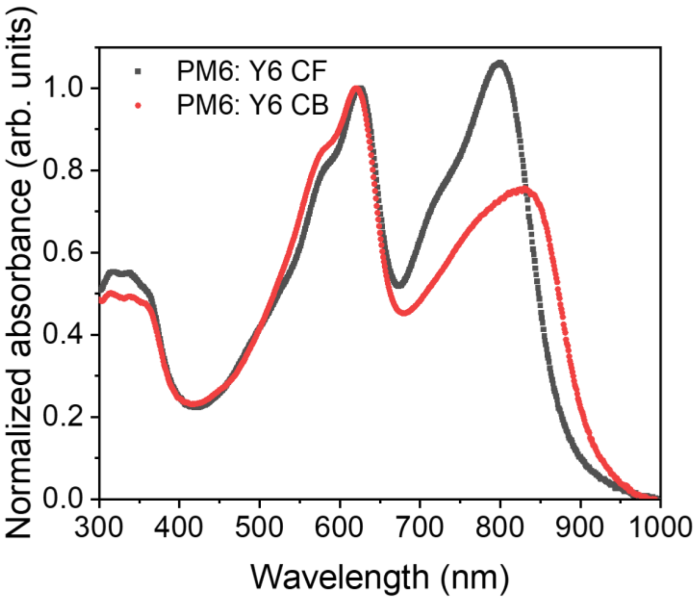

Supplementary Fig. 18. Absorbance spectra of bilayer films. The absorption spectra of PM6:Y6 CF and CB blend films normalized with respect to the PM6 absorption peak.

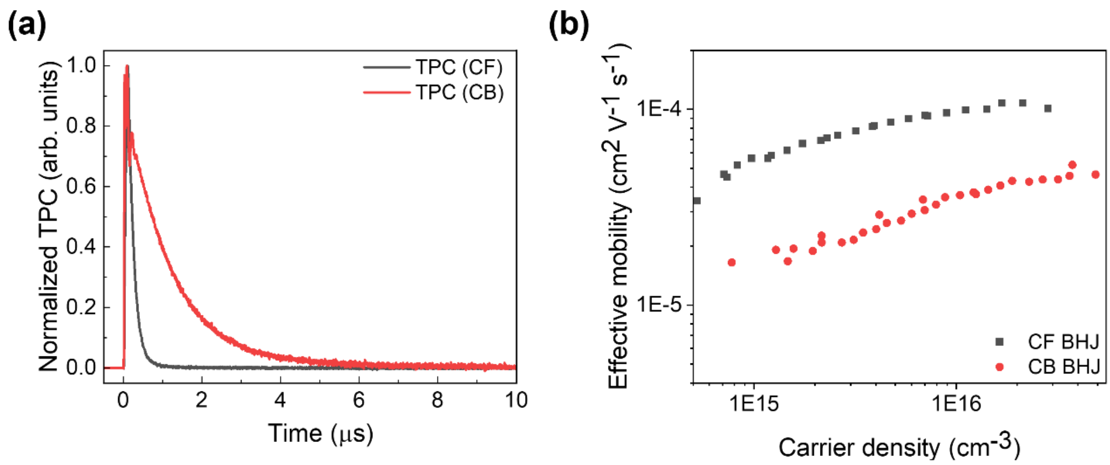

Supplementary Fig. 19. Charge carrier extraction in bilayer films. (a) Transient photocurrent (TPC) decay of PM6:Y6 CF and CB BHJs measured under 1 sun and short-circuit condition. The slower photocurrent transient observed for the CB BHJ indicates slower charge extraction. **(b)** Effective carrier mobilities for PM6: Y6 CF and CB BHJs as a function of carrier density derived from TPV & TPC measured under a range of light intensities.

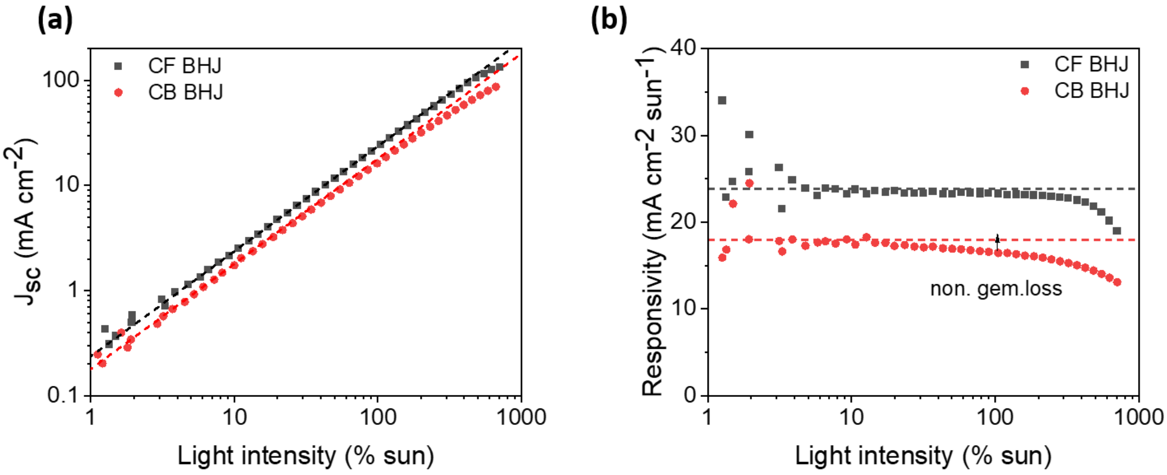

Supplementary Fig. 20. Light intensity-dependent measurements of BHJ devices. (a) Intensity-dependent J_{SC} measurements for PM6:Y6 CF and CB BHJs and **(b)** the corresponding responsivity obtained by dividing J_{SC} by the light intensity. The saturated values at low light intensity are highlighted by the dashed lines. The decrease in the responsivity with increasing light intensity indicates the presence of non-geminate recombination loss under the short-circuit condition. The arrow highlights the higher loss in the CB BHJ under 1 sun.

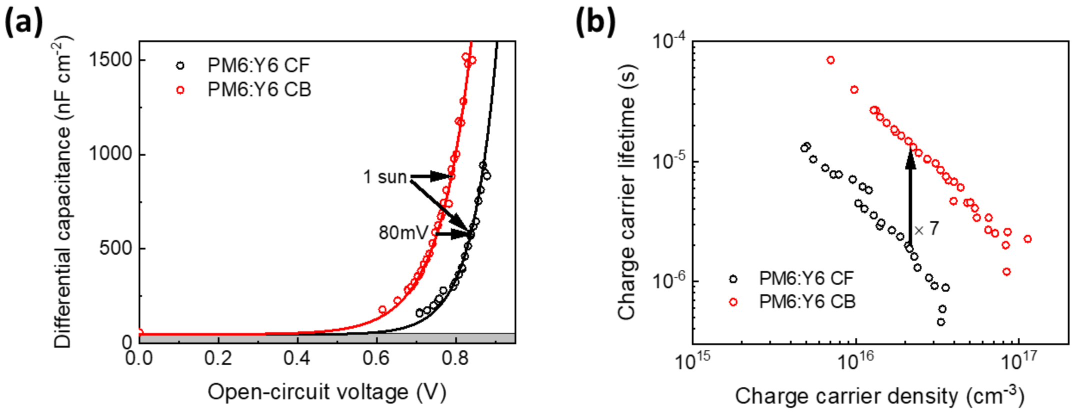

Supplementary Fig. 21. Transient optoelectronic characterizations of BHJ devices. (a) Differential capacitance vs. open-circuit voltage and **(b)** charge carrier lifetime determined from TPV vs. the spatially averaged charge carrier density determined from the differential charging method of the PM6:Y6 BHJ devices processed by CF and CB. The difference in charge carriers stored in the active layer under 1 sun illumination and the shift in the electronic bandgap are highlighted in (a).

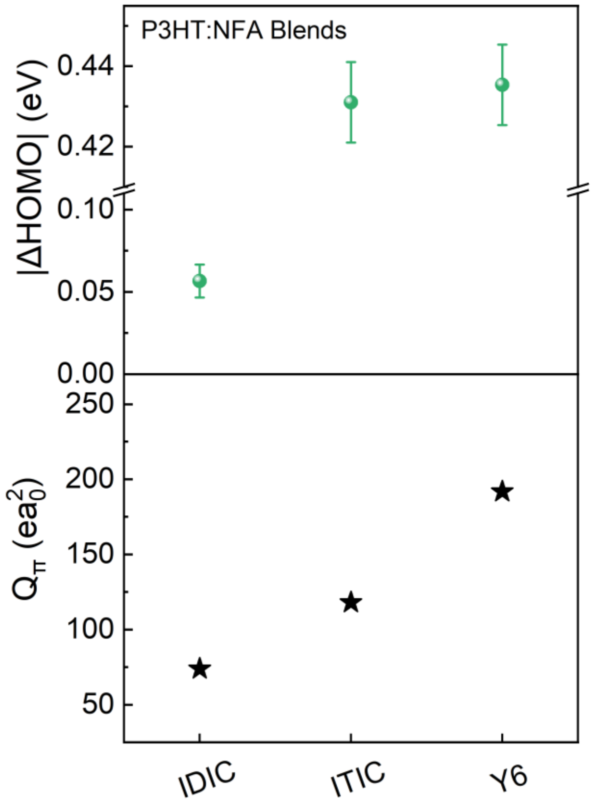

Supplementary Fig. 22. Energetics of P3HT:NFA blend films. Quadrupole effect on HOMO level shift (ΔHOMO) of P3HT upon blending with different Q_π NFAs of IDIC, ITIC and Y6. Similar to PM6 and PBDB-T cases, P3HT:NFA BHJs also exhibit a positive correlation with ΔHOMO and NFA's Q_π values. (Error bars are the intrinsic machine error.)
