# Supplementary material for: Molecular orientation-dependent energetic shifts in solution-processed non-fullerene acceptors and their impact on organic photovoltaic performance
Source: Nat Commun. 2023 Apr 4;14:1870. doi: 10.1038/s41467-023-37234-0 (PMC10073232; doi:10.1038/s41467-023-37234-0)
Supplement: Supplementary file 2 — Description of Additional Supplementary Files [file 41467_2023_37234_MOESM2_ESM.pdf]

File name: Supplementary Data 1

Description: Molecular dynamic initial configuration

File name: Supplementary Data 2

Description: Molecular dynamic final configuration

File name: Supplementary Data 3

Description: DFT configuration for edge-on oriented Y6

File name: Supplementary Data 4

Description: DFT configuration for face-on oriented Y6
