# Supplementary material for: Molecular orientation-dependent energetic shifts in solution-processed non-fullerene acceptors and their impact on organic photovoltaic performance
Source: Nat Commun. 2023 Apr 4;14:1870. doi: 10.1038/s41467-023-37234-0 (PMC10073232; doi:10.1038/s41467-023-37234-0)
Supplement: Supplementary file 7 — Solar Cells Reporting Summary [file 41467_2023_37234_MOESM7_ESM.pdf]

nature research

Corresponding author(s): Ji-Seon Kim

# Solar Cells Reporting Summary

Nature Research wishes to improve the reproducibility of the work that we publish. This form is intended for publication with all accepted papers reporting the characterization of photovoltaic devices and provides structure for consistency and transparency in reporting. Some list items might not apply to an individual manuscript, but all fields must be completed for clarity.

For further information on Nature Research policies, including our data availability policy, see Authors & Referees.

## Experimental design

**Please check: are the following details reported in the manuscript?**

### 1. Dimensions

Area of the tested solar cells — [x] Yes [ ] No — Methods section in the main manuscript

Method used to determine the device area — [x] Yes [ ] No — Methods section in the main manuscript

### 2. Current-voltage characterization

Current density-voltage (J-V) plots in both forward and backward direction — [ ] Yes [x] No — Only forward scan reported as solar cells are not affected by any hysteresis effect

Voltage scan conditions
*For instance: scan direction, speed, dwell times* — [ ] Yes [x] No — Negligible effects on J-V curves

Test environment
*For instance: characterization temperature, in air or in glove box* — [x] Yes [ ] No — Methods section in the main manuscript

Protocol for preconditioning of the device before its characterization — [ ] Yes [x] No — Negligible effects on J-V curves

Stability of the J-V characteristic
*Verified with time evolution of the maximum power point or with the photocurrent at maximum power point; see ref. 7 for details.* — [ ] Yes [x] No — Not applicable for the scope of the manuscript

### 3. Hysteresis or any other unusual behaviour

Description of the unusual behaviour observed during the characterization — [ ] Yes [x] No — Not applicable for the scope of the manuscript

Related experimental data — [ ] Yes [x] No — Not applicable

### 4. Efficiency

External quantum efficiency (EQE) or incident photons to current efficiency (IPCE) — [x] Yes [ ] No — Results section in the main manuscript

A comparison between the integrated response under the standard reference spectrum and the response measure under the simulator — [ ] Yes [x] No — We didn't deem this information relevant in the scope of the study, although the response is comparable

For tandem solar cells, the bias illumination and bias voltage used for each subcell — [ ] Yes [x] No — Not applicable

### 5. Calibration

Light source and reference cell or sensor used for the characterization — [x] Yes [ ] No — Methods section in the main manuscript

Confirmation that the reference cell was calibrated and certified — [x] Yes [ ] No — Methods section in the main manuscript

Calculation of spectral mismatch between the reference cell and the devices under test

- [ ] Yes
- [x] No

Not applicable

6. Mask/aperture

Size of the mask/aperture used during testing

- [ ] Yes
- [x] No

No mask is used

Variation of the measured short-circuit current density with the mask/aperture area

- [ ] Yes
- [x] No

No mask is used

7. Performance certification

Identity of the independent certification laboratory that confirmed the photovoltaic performance

- [ ] Yes
- [x] No

Not applicable

A copy of any certificate(s)
*Provide in Supplementary Information*

- [ ] Yes
- [x] No

Not applicable

8. Statistics

Number of solar cells tested

- [ ] Yes
- [x] No

Not applicable (no statistics analysis provided)

Statistical analysis of the device performance

- [ ] Yes
- [x] No

Not applicable (no statistics analysis provided)

9. Long-term stability analysis

Type of analysis, bias conditions and environmental conditions
*For instance: illumination type, temperature, atmosphere humidity, encapsulation method, preconditioning temperature*

- [ ] Yes
- [x] No

Not applicable
